# New Progress on London Dispersive Energy, Polar Surface Interactions, and Lewis’s Acid–Base Properties of Solid Surfaces

**DOI:** 10.3390/molecules29050949

**Published:** 2024-02-21

**Authors:** Tayssir Hamieh

**Affiliations:** 1 Faculty of Science and Engineering, Maastricht University, P.O. Box 616, 6200 MD Maastricht, The Netherlands; t.hamieh@maastrichtuniversity.nl; Tel.: +31-6-5723-9324; 2 Laboratory of Materials, Catalysis, Environment and Analytical Methods (MCEMA), Faculty of Sciences, Lebanese University, Hadath P.O. Box 6573, Lebanon

**Keywords:** London dispersive energy, polar energy of adsorption, polar enthalpy and entropy of adsorption, enthalpic and entropic Lewis’s acid–base parameters, separation distance between particles, acid–base surface energy

## Abstract

The determination of the polar surface free energy, polar properties, and Lewis’s acid base of solid materials is of capital importance in many industrial processes, such as adhesion, coatings, two-dimensional films, and adsorption phenomena. (1) Background: The physicochemical properties of many solid particles were characterized during the last forty years by using the retention time of injected well-known molecules into chromatographic columns containing the solid substrates to be characterized. The obtained net retention time of the solvents adsorbed on the solid, allowing the determination of the net retention volume directly correlated to the specific surface variables, dispersive, polar, and acid–base properties. (2) Methods: Many chromatographic methods were used to quantify the values of the different specific surface variables of the solids. However, one found a large deviation between the different results. In this paper, one proposed a new method based on the London dispersion equation that allowed the quantification of the polar free energy of adsorption, as well as the Lewis’s acid–base constants of many solid surfaces. (3) Results: The newly applied method allowed us to obtain the polar enthalpy and entropy of adsorption of polar model organic molecules on several solid substrates, such as silica, alumina, MgO, ZnO, Zn, TiO_2_, and carbon fibers. (4) Conclusions: our new method based on the separation between the dispersive and polar free surface energy allowed us to better characterize the solid materials.

## 1. Introduction

Dispersion and polar interactions are the two important types of interactions between particles. The determination of these interactions is very often used in the different domains of colloidal science, surface physics, adsorption, adhesion, surface, and interface. The dispersive interactions were studied and well developed by Van der Waals. The corresponding forces, called Van der Waals forces, result from the temporary fluctuations in the charge distribution of the atoms or molecules; whereas, the polar forces or interactions include Coulomb interactions between permanent dipoles and between permanent and induced dipoles. The total interaction energy is the sum of the dispersive and polar interaction energies. The separation of these two types of energy is crucial to understanding the behavior of molecules and, therefore, to predicting the various surface physicochemical properties of materials and nanomaterials.

Since 1982, many scientists proposed several methods to separate the dispersive (or London) and polar (or specific) interactions between a solid substrate and a polar molecule. The first attempt for the separation of the two above contributions was proposed by Saint-Flour and Papirer [1,2,3] when studying untreated and silane-treated glass fibers by using inverse gas chromatography (IGC) and choosing a series of polar and non-polar adsorbates to quantify the dispersive and polar free energies. The authors adopted the concept of the vapor pressure $P_{0}$ of the adsorbates to determine the specific free energy of adsorption $∆G_{a}^{sp}(T)$ of polar molecules on glass fibers as a function of the absolute temperature *T* by plotting the variations of RTlnVn versus the logarithm of the vapor pressure $P_{0}$ of the probe, where Vn is the net retention volume and *R* the ideal gas constant. Saint-Flour and Papirer [3] determined the specific enthalpy $∆H_{a}^{sp}$ and entropy $∆S_{a}^{sp}$ of polar molecules adsorbed on the glass fibers and deduced their Lewis acid–base constants. Later, Schultz et al. [4] tried to separate the two dispersive and specific interactions of carbon fibers by using the concept of the dispersive component $\gamma_{l}^{d}$ of the surface energy of the organic liquids by drawing RTlnVn as a function of the $2Na\;\sqrt{\gamma_{l}^{d}}\;$of n-alkanes and polar molecules adsorbed on the solid, where *a* is the surface area of the adsorbed molecule and N is the Avogadro’s number. This method allowed them to obtain the specific free energy and the dispersive component $\gamma_{s}^{d}$ of the surface energy of carbon fibers. In 1991, Donnet et al. [5]. used the deformation polarizability $\alpha_{0,L}$ of solvents and obtained the specific free energy $∆G_{a}^{sp}(T)$ of polar solvents adsorbed on natural graphite powders by representing the variations as a function of ${\sqrt{h\nu_{L}}\;\alpha }_{0,L}$, where $\nu_{L}$ is the electronic frequency of the probe and *h* is the Planck’s constant. With the difficulties and issues encountered with the previous methods, Brendlé and Papirer [6,7] used the topological index $\chi_{T}$, derived from the well-known Wiener index to obtain more accurate results. Other methods were also used in the literature, such as the boiling point $T_{B.P.}$ [8] and the standard enthalpy of vaporization $∆H_{vap.}^{0}$ [9]. In all the above methods, one obtained excellent linearity of the RTlnVn of n-alkanes as a function of the chosen intrinsic thermodynamic parameter (${lnP}_{0}$, $a\;\sqrt{\gamma_{l}^{d}}$, ${\sqrt{h\nu_{L}}\;\alpha }_{0,L}$, $T_{B.P.}$ or $∆H_{vap.}^{0}$). The specific free potential $∆G_{a}^{sp}(T)$ of a polar molecule is then directly obtained by the distance from the point representing the polar molecule to its hypothetic point located on the n-alkane straight line. The specific enthalpy and entropy of adsorbed polar solvents, as well as the Lewis acid–base constants, can be easily deduced by thermodynamic considerations. The determination of these surface properties is of capital importance in the different industrial and practical domains implying adhesion, fusion, adsorption, desorption, or contact between surfaces and interfaces of solid materials, which vary from organic, inorganic, or food materials; porous; polymers or copolymers; pharmaceutical; carbon black; ceramics; to metallic oxides in powder or fiber form [10,11,12,13,14,15,16,17,18,19,20,21,22,23,24,25,26,27].

The serious problem encountered in these different chromatographic methods is that the obtained results cannot be considered quantitative and can only give a qualitative comparison at most between solid materials. The wrong determination of the surface thermodynamic parameters of interaction between materials or nanomaterials and the probe molecules will be catastrophic for an accurate prediction of the reactivity, work of adhesion, or contact between these materials. This required finding more confident methods to better catheterize the different types of interactions and their behavior with other molecules.

One proved in several previous studies the non-validity of the method used by Schultz et al. due to the variations of the surface area *a* and $\gamma_{l}^{d}$ of solvents as a function of the temperature [28,29,30,31,32]. The values of the surface area of organic molecules versus the temperature obtained on a certain solid material [28,29,30,31,32] cannot always be transferred to another solid because of the different behaviors existing between the various solid surfaces and the adsorbed molecules.

The used chromatographic methods, even if they satisfied linear relations for n-alkanes adsorbed on solid surfaces, cannot be necessarily considered accurate if they are not theoretically well founded. One proved in a previous paper [29,30] that the linearity of the RTlnVn of n-alkanes is satisfied for more than twenty intrinsic thermodynamic parameters and one concluded on the necessity to find new methods that are theoretically valid. 

Given the disparity of the results obtained from the application of the various methods, one privileged, in this paper, the method based on the equation of the London dispersive interaction [33] between the solvents and the solid materials. Indeed, the only concept well founded theoretically is the London equation that exactly quantifies the dispersive interaction between particles and solid surfaces by considering the important notion of polarizabilities of organic molecules and materials. By using the London equation [33], one proposed, in this study, to determine the dispersive free energy $∆G_{a}^{d}$, the specific free energy $∆G_{a}^{sp}$, the Lewis acid–base constants, and the polar acidic and basic surface energy of several solid materials, such as silica (SiO_2_), alumina (Al_2_O_3_), magnesium oxide (MgO), zinc oxide (ZnO), Monogal-Zn, titanium dioxide (TiO_2_), and carbon fibers. This method gave more accurate values of the dispersive and polar interactions between the above solid surfaces and the different organic solvents and correct Lewis acid–base properties of the various solids.

## 2. Results

### 2.1. New Approach for the Calculation of the Deformation Polarizability $\alpha_{0X}$ and the Indicator Parameter $P_{SX}$

Our new approach, previously presented, allowed us to obtain all necessary parameters of organic solvents and solid substrates by using their values taken from the Handbook of Physics and Chemistry [34]. The obtained results are presented below in Table 1, Table 2, Table 3, Table 4, Table 5, Table 6, Table 7 and Table 8.

The new values of the various parameters given in Table 1, Table 2, Table 3, Table 4, Table 5, Table 6, Table 7 and Table 8 were used in our new method to give the new values of the London dispersive and polar energies of the various solid materials.

In this new approach, one gave more precise values of the parameters of molecules, such as the deformation polarizability and the harmonic mean of the ionization energies of solids and organic solvents, contrary to those proposed by Donnet et al. [5] that only took the characteristic electronic frequencies of the probes independently of those of the solid. Indeed, Table 2, Table 3, Table 4, Table 5, Table 6, Table 7 and Table 8 clearly show that the harmonic mean of the ionization energies of the solvents varied as a function of the used solid material.

To show the difference between our values and those of Donnet et al. [5], one presented, in Table 9, the values of the deformation polarizability of some polar molecules and two n-alkanes.

Table 9 shows that the values relative to some solvents, such as n-decane, dichloromethane, and methanol, and those of solid particles are not given by Donnet et al. The relative error reaches 10%, which can have a negative effect on the determination of the specific free energy. 

Now, if one adds the error committed by Donnet et al. [5] when neglecting the variations of the harmonic mean of ionization energies $\frac{\varepsilon_{S}\;\varepsilon_{X}}{\left(\varepsilon_{S}\;+\;\varepsilon_{X}\right)}$ for the various polar molecules that vary from 20% to 70%, indeed, this parameter varies from a solid surface to another solid material. The variation in the value of the $\frac{\varepsilon_{S}\;\varepsilon_{X}}{\left(\varepsilon_{S}\;+\;\varepsilon_{X}\right)}$ of organic molecules between two solids can reach 70% in certain cases, such as ZnO and TiO_2_ (Table 4 and Table 7).

### 2.2. London Dispersive Surface Energy of Solid Particles by Using the Thermal Model

The thermal model [28,29,30,31,32] was used to determine the London dispersive surface energy $\gamma_{s}^{d}\;(T)$ of the various solid materials used in this study. This model took into consideration the effect of the temperature on the surface area of organic molecules. The obtained results are presented in Table 10 at several temperatures.

Table 10 shows that the various solid surfaces can be classified with increasing order of their London dispersive surface energy as follows:

Oxidized carbon fibers < Untreated carbon fibers < MgO < ZnO < Al_2_O_3_ < Monogal-Zn < SiO_2_

**Figure 1:**
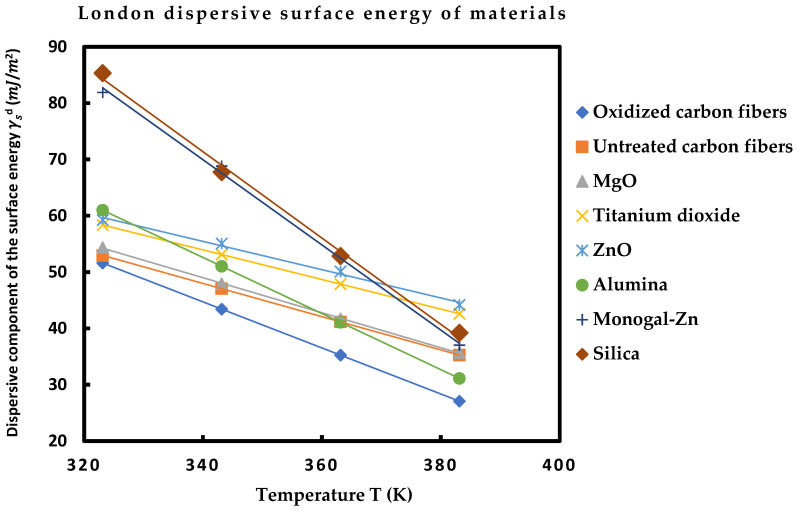
Dispersive component of the surface energy $\gamma_{s}^{d}\;(mJ/m^{2})$ of solid materials as a function of the temperature *T* (K).

The highest London dispersive surface energy was obtained by the silica particles. One also observed that the dispersive surface energies of the two carbon fibers are very close and the silica and monogal surfaces exhibited close values of $\gamma_{s}^{d}$. Furthermore, the linearity of $\gamma_{s}^{d}\;(T)$ was assured for all materials with excellent linear regression coefficients approaching 1.000 (Figure 1).

### 2.3. Polar Surface Interactions between Solid Materials and Organic Molecules 

By using our new method and new findings presented in Section 3, one determined the values of the polar free surface energy ($-∆G_{a}^{sp}\left(T\right)$) of the various polar solvents adsorbed on the various solid particles as a function of the temperature *T*. The results are given in Table 11.

Table 11 clearly shows the amphoteric behavior of the various solid surfaces with different acid–base interactions depending on the number of the surface group sites present on the solid particles. Table 11 led to the classification of the polar solvents for each solid surface in increasing order of the polar free surface energy of the interaction.

In the case of silica particles, one obtained the following order:

Ethyl Acetate < CCl_4_ < Acetone < Nitromethane < Toluene < CHCl_3_ < CH_2_Cl_2_ < Diethyl ether < THF

Proving a strong interaction with the acidic organic molecules and a lower one with the basic solvents led to concluding more basic behavior.

In this case of MgO, the obtained order was:

CH_2_Cl_2_ < CHCl_3_ < Ethyl acetate < Diethyl ether < Acetone < Tetrahydrofuran

That showed a behavior that was rather amphoteric.

For ZnO, one also observed a strong amphoteric character:

Benzene < CHCl_3_ < CH_2_Cl_2_ < Ethyl acetate < Diethyl ether < Tetrahydrofuran

The amphoteric character was proved for monogal-Zn particles:

CH_2_Cl_2_ < Ethyl acetate < CHCl_3_ < Diethyl ether < Acetone < Tetrahydrofuran

For alumina, one obtained the following order:

CCl_4_ < CH_2_Cl_2_ < Ethyl acetate < Diethyl ether < CHCl_3_ < Toluene < Tetrahydrofuran

In the case of TiO_2_:

CH_2_Cl_2_ < CHCl_3_ < Ethyl acetate < Acetonitrile < Benzene < Acetone < THF < nitromethane

For untreated carbon fibers:

CCl_4_ < Diethyl ether < CH_2_Cl_2_ < Benzene < Ethyl acetate < Tetrahydrofuran 

And the oxidized carbon fibers presented an amphoteric character:

CCl_4_ < Diethyl ether < Benzene < CH_2_Cl_2_ < CHCl_3_ < Benzene < Ethyl acetate < THF < Acetone 

**Figure 2:**
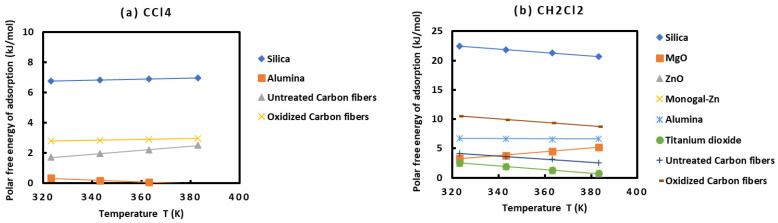

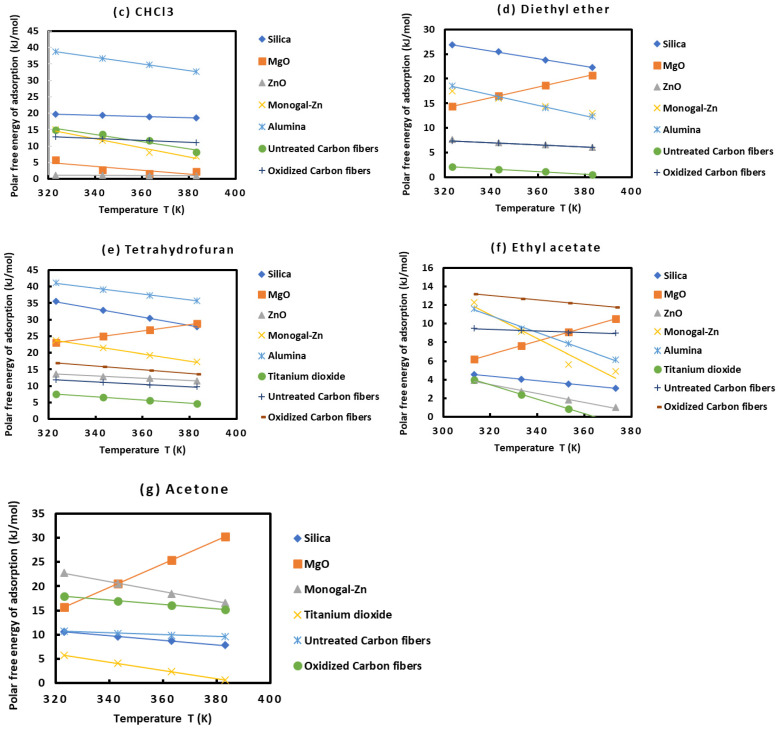
Evolution of the specific free surface energy ($-∆G_{a}^{sp}\left(T\right)$) of the various solid materials in interactions with the different polar molecules, such as CCl_4_ (**a**), CH_2_Cl_2_ (**b**), CHCl_3_ (**c**), diethyl ether (**d**), tetrahydrofuran (**e**), ethyl acetate (**f**), and acetone (**g**), as a function of the temperature.

In order to compare the behavior of the various solids as a function of the different polar solvents, one plotted in Figure 2 the variations of the ($-∆G_{a}^{sp}\left(T\right)$) of the various polar molecules as a function of the temperature.

The results in Figure 2 show different behaviors of the various solid surfaces in interaction with the polar molecules. One gave the classification of these solid materials in increasing order of their polar free energies with the different polar solvents:

○ With CCl_4_: alumina < untreated carbon fibers < oxidized carbon fibers < silica;
○ With CH_2_Cl_2_: Monogal-Zn < ZnO < TiO_2_ < MgO < untreated carbon fibers < alumina < oxidized carbon fibers < silica;
○ With CHCl_3_: ZnO < MgO < oxidized carbon fibers < untreated carbon fibers < Monogal-Zn < silica < alumina;
○ With diethyl ether: untreated carbon fibers < oxidized carbon fibers < ZnO < MgO < Monogal-Zn < alumina < silica;
○ With tetrahydrofuran: TiO_2_ < untreated carbon fibers < ZnO < oxidized carbon fibers < MgO < Monogal-Zn < silica < alumina;
○ With ethyl acetate: TiO_2_ < ZnO < silica < MgO < untreated carbon fibers < alumina < monogal-Zn < oxidized carbon fibers;
○ With acetone: TiO_2_ < silica < untreated carbon fibers < MgO < oxidized carbon fibers.

These results proved that alumina, silica, and oxidized carbon fibers exhibited stronger interactions with the acidic and basic molecules, showing their higher amphoteric character than the other solid substrates.

### 2.4. Lewis’s Enthalpic and Entropic Acid–Base Parameters 

By using the results of $∆G_{a}^{sp}\left(T\right)$ given in Table 11 and Figure 2, one determined, from Equation (11), the different values of the polar enthalpy ($-∆H_{a}^{sp})$ and entropy ($-∆S_{a}^{sp})$ of the adsorption of the various polar molecules on the solid surfaces. The results are presented in Table 12.

Table 12 also shows a difference in the behavior of the various solid surfaces in interactions with acidic, basic, and amphoteric polar solvents. The acid–base constants of the solid materials were calculated. The obtained values of the Lewis enthalpic acid–base constants $K_{A}$ and $K_{D}\;$and the Lewis entropic acid–base constants $\omega_{A}$ and $\omega_{D}\;$of the different solid particles are presented in Table 13. The comparison of the acid–base behavior of the different solid materials allowed us to classify them in decreasing order of acidity and basicity.

For the acidity, one obtained the following classification:

Silica > alumina > Monogal-Zn > TiO_2_ > ZnO > oxidized carbon fibers > untreated carbon fibers > MgO 

Whereas, the comparison between their basicity led to give the following order:

Oxidized carbon fibers > alumina > untreated carbon fibers > ZnO > Monogal-Zn > Silica > MgO > TiO_2_

By comparing the various solids in decreasing order of their ratio $K_{D}$/$K_{A}$, one found the following classification:

Oxidized carbon fibers > untreated carbon fibers > MgO > ZnO > TiO_2 >_ alumina > Monogal-Zn > Silica

The last classification seems to be very interesting because the oxidization of carbon fibers will increase the polar surface groups and, therefore, their basicity, contrary to the behavior of silica, which exhibits higher acidity than the other solid surfaces.

However, when we observed the linear regression coefficients given in Table 13, we found that the linearity of Equations (13) and (23) are not satisfied for most of the solid surfaces. In such a case, a correction has to be executed. To do that, one used Equation (17) and resolved the linear system with three unknown numbers. The solution was performed for all solids, except for titanium dioxide, which presented an excellent linear regression coefficient. More results are given in Table 14.

Table 14 gives the corrected values of the acid–base constants with an additional constant called a coupling constant reflecting the amphoteric character of materials.

One observed that the classification of acidity of different solid materials was conserved after correction; however, it was changed for the basicity. One found the following classification of solid surfaces in decreasing basicity:

Oxidized carbon fibers > silica > monogal-Zn > untreated carbon fibers > alumina > ZnO > TiO_2_ > MgO

It was proved that the oxidized carbon fibers exhibited the strongest basicity whereas silica had the highest acidity. It was also shown that the MgO presented a more neutral surface with a small basic tendency.

**Figure 3:**
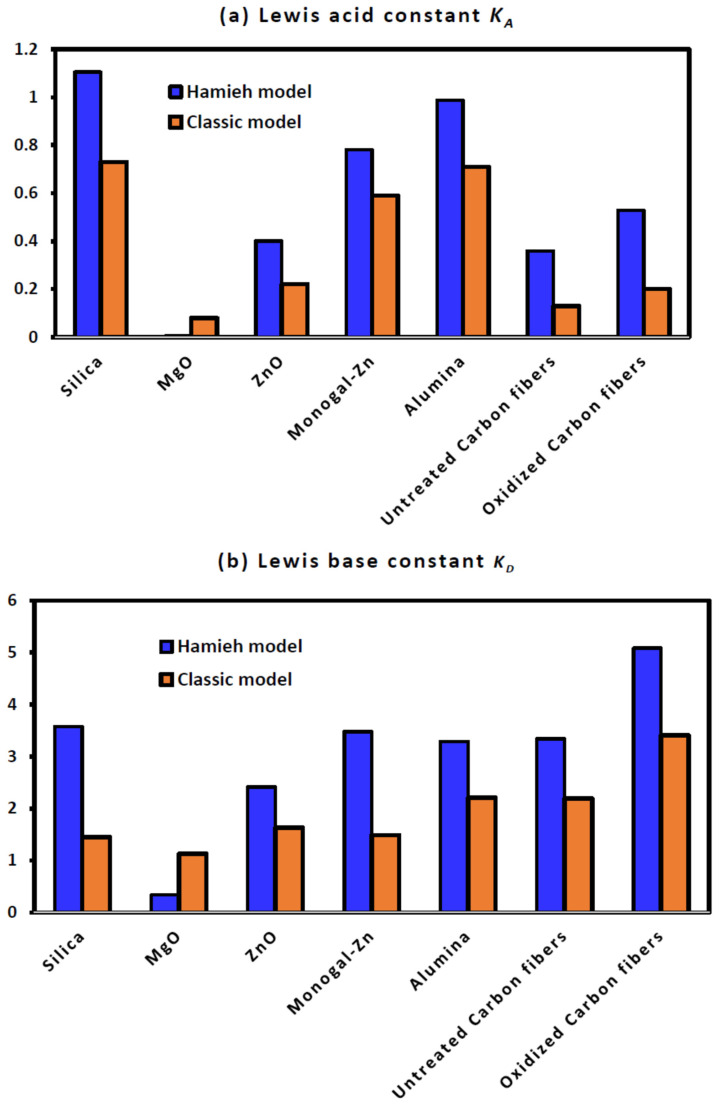

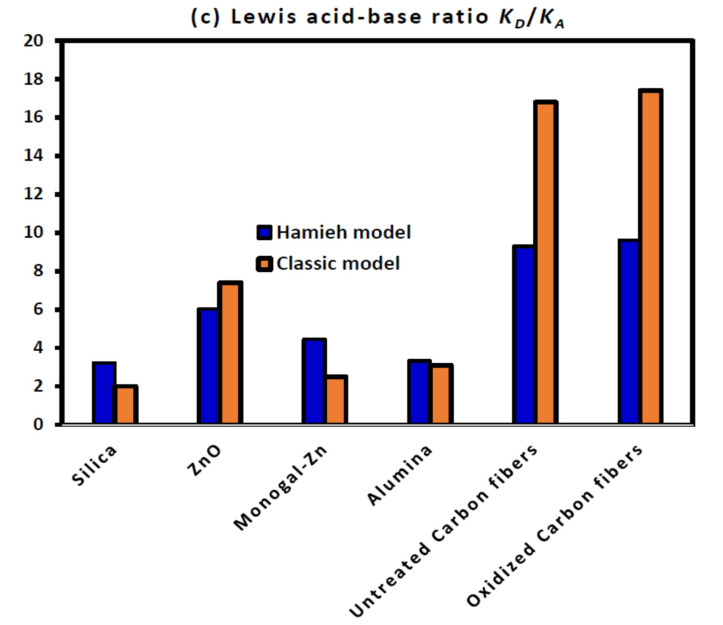
Comparison between the Lewis acid–base constants of the various solid substrates. (**a**) acid constant, (**b**) base constant, and (**c**) acid–base ratio.

The comparison between the values of Lewis’s acid–base constants obtained by the classic method and those corrected by using the Hamieh model is shown in Figure 3 for the various solid materials.

Figure 3a,b show that the classic method underestimated the values of Lewis acid and base constants of the different solid materials with respect to those obtained by the Hamieh model. The deviation reached 50% in some cases. This also affected the Lewis acid–base ratio, which was exaggerated by the classic method (Figure 3c) for the case of the two carbon fibers. A special case was obtained with magnesium oxide with very small values of the acid–base constants. Indeed, the acid constant of MgO is approximately equal to zero and a negative value of the amphoteric constant was obtained by the Hamieh model, proving that this solid material exhibited a very weak amphoteric character and can be used as an inert material. 

### 2.5. Consequences of the Application of the New Method

The first scientific result of the application of the new parameter $P_{SX}=\frac{\varepsilon_{S}\;\varepsilon_{X}}{\left(\varepsilon_{S}\;+\;\varepsilon_{X}\right)}\alpha_{0X}$ relative to the interaction between solids and organic molecules was the separation between the London dispersive energy and the polar free energy of the adsorption of polar organic molecules and solid surfaces. It is the first time that we were able to calculate exactly the two contributions of the free surface energy of the interaction. Equation (6) was perfectly applied for all solids and solvents with an excellent linear regression coefficient approaching 1.000 and the determination of the slope labeled A of the straight line given by Equation (6) in the case of the n-alkanes adsorbed on solid surfaces was conducted to calculate the London dispersive energy of the interaction not only for n-alkanes but also for polar organic solvents by using the following relation:

(1) $$∆G_{a}^{d}\left(T\right)=A\left[\frac{3N}{{2\left(4\pi \varepsilon_{0}\right)}^{2}}P_{SX}\right]$$

With this new approach, one characterized all studied solids given in Tables S1–S16 (See the Supplementary Materials), the two London dispersive and polar free energies of the interaction between solids and organic molecules. This also allowed us to obtain the total free surface energy of adsorption without calculating the surface-specific area of the considered solid materials.

The second consequence was to clearly verify the insufficiency of the approach proposed by Donnet et al. [5]. Indeed, if we applied their method to silica particles, one obtained the values of the ($-∆G_{a}^{sp}\left(T\right)$) of the polar solvents adsorbed on silica surfaces. These results compared to our new findings are presented in Table 15.

The results in Table 15 clearly show a large difference between the values obtained by the two above methods. The calculation of the ratios $\frac{\left(-∆G_{a}^{sp}\left(Donnet\;et\;al.\right)\right)}{\left(-∆G_{a}^{sp}\left(Hamieh\right)\right)}$, $\frac{\left(-∆S_{a}^{sp}\left(Donnet\;et\;al.\right)\right)}{\left(-∆S_{a}^{sp}\left(Hamieh\right)\right)}$, and $\frac{\left(-∆H_{a}^{sp}\left(Donnet\;et\;al.\right)\right)}{\left(-∆H_{a}^{sp}\left(Hamieh\right)\right)}$ given in Table 16 showed a surestimation of the values of $\left(-∆G_{a}^{sp}\left(T\right)\right)$ obtained by the Donnet et al. method, varying from 1.3 to 7.7 times the values obtained by our new method. Whereas, in the calculation of the specific entropy and enthalpy, Table 16 shows ratios varying from 3.1 to 23.7, strongly depending on the adsorbed polar molecule. However, one globally found a ratio approaching 2 for most polar molecules. 

These large variations of the values obtained by applying the Donnet et al. method are certainly due to the fact that this method omitted the variation of the harmonic mean $\bar{\varepsilon_{S-X}}$ of the ionization energies of the solid and the adsorbed polar solvent given by relation (2):

(2) $$\bar{\varepsilon_{S-X}}=\frac{\varepsilon_{S}\;\varepsilon_{X}}{\left(\varepsilon_{S}+\varepsilon_{X}\right)}$$

Donnet et al. used the concept $\alpha_{0}\sqrt{\nu_{0}}$ or $\alpha_{0X}\sqrt{\varepsilon_{X}}$. The variations of $\bar{\varepsilon_{S-X}}$ are not identical to those of the $\sqrt{\varepsilon_{X}}$ of the interaction solid-polar molecule as it is shown in Table 17.

It can be observed in Table 17 that the harmonic mean $\bar{\varepsilon_{S-X}}$ strongly depends on the interaction between the solid and the polar solvent and cannot be considered as constant for all studied materials, as was supposed by the method proposed by Donnet et al.

The third consequence of our new approach was the determination of the average separation distance H between the solid particle and the organic molecule as a function of the temperature when the deformation polarizability of the solid is known. By using Equation (7) and the experimental results, one presents, in Table 18, the values of the average separation distance H at different temperatures for the various solid substrates.

Table 18 shows that the average separation distance H is comprised between 4.45 Å and 5.56 Å for the various solid particles. A slight increasing effect of the temperature on the separation distance was observed in all studied solid substrates. Furthermore, one observed that the separation distance between a solid and an organic molecule is an intrinsic parameter of the solid. Table 18 allows us to classify the various solid materials in increasing order of the separation distance for all temperatures:

Untreated carbon fibers ≈ Oxidized carbon fibers > ZnO > alumina > Monogal-Zn > Silica > MgO > TiO_2_

This classification is very close to that obtained with the basicity of solid materials. It seems that when the basicity or the ratio $K_{D}$***/***$K_{A}$ decreases, the separation distance slightly increases to reach a maximum value with TiO_2_ equal to 5.50 Å.

The fourth consequence of this new method was to be able to give, with more accuracy, the values of the acid–base surface energy of the various solid materials. Indeed, this was obtained by applying the Van Oss et al. relation [35] that gave the specific enthalpy of adsorption as a function of the Lewis acid surface energy of the solid surface $\gamma_{s}^{+}$ and the solvent $\gamma_{l}^{+}$ and the corresponding Lewis base surface energy ($\gamma_{s}^{-}$ for the surface and $\gamma_{l}^{-}$ for the solvent) by Equation (3):

(3) $$∆G_{a}^{sp}\left(T\right)=2Na\left(\sqrt{\gamma_{l}^{-}\gamma_{s}^{+}}+\sqrt{\gamma_{l}^{+}\gamma_{s}^{-}}\right)$$

By choosing two monopolar solvents, such as ethyl acetate (EA) and dichloromethane, characterized by:

(4) $$\left\{\begin{array}{c} \gamma_{CH2Cl2}^{+}=5.2\;mJ/m^{2}\;,\;\;\gamma_{CH2Cl2}^{-}=0\\ \gamma_{EA}^{+}=0\;,\;\;\;\;\;\;\;\;\;\;\;\;\gamma_{EA}^{-}=19.2\;mJ/m^{2} \end{array}\right.$$

The Lewis acid and base surface energies of a solid surface $\gamma_{s}^{+}$ and $\gamma_{s}^{-}$ can be obtained from Relations (3) and (4):

(5) $$\left\{\begin{array}{c} \begin{array}{c} \gamma_{s}^{+}=\frac{{\left[∆G_{a}^{sp}\left(T\right)\left(EA\right)\right]}^{2}}{{4N}^{2}{\left[a\left(EA\right)\right]}^{2}\gamma_{EA}^{-}}\; \end{array}\;\;\;\;\;\;\;\;\;\;\;\;\;\;\\ \gamma_{s}^{-}=\frac{{\left[∆G_{a}^{sp}\left(T\right)\left(CH2Cl2\right)\right]}^{2}}{{4N}^{2}{\left[a\left(CH2Cl2\right)\right]}^{2}\gamma_{CH2Cl2}^{+}}\; \end{array}\right.$$

With the experimental values of the free specific energy of ethyl acetate $∆G_{a}^{sp}\left(T\right)\left(EA\right)$ and dichloromethane $∆G_{a}^{sp}\left(T\right)\left(CH2Cl2\right)$ given in Table 19, one determined the values of the specific acid and base surface energy contributions $\gamma_{s}^{+}$, $\gamma_{s}^{-}$, as well as the acid–base surface energy $\gamma_{s}^{AB}$ given by Relation (6):

(6) $$\gamma_{s}^{AB}=2\sqrt{\gamma_{s}^{+}\gamma_{s}^{-}}$$

By using the values given in Table 10 and Table 19 and Relation (6), one presented, in Table 20, Lewis’s acid and base surface energies of solid particles $\gamma_{s}^{+}$, $\gamma_{s}^{-}$, $\gamma_{s}^{AB}$ and the total surface energy $\gamma_{s}^{tot.}$ of the various solid materials. The total surface energy $\gamma_{s}^{tot.}$ of the solid surfaces was obtained by using Relation (7):

(7) $$\gamma_{s}^{tot.}={\gamma_{s}^{d}+\gamma }_{s}^{AB}$$

The values of the dispersive surface energy of the different solid materials were taken from Table 10.

The values of the different acid–base surface energies of the various solid substrates given in Table 20 showed that the oxidized carbon fibers and the silica particles gave the highest values of $\gamma_{s}^{-}$, $\gamma_{s}^{AB}$, and $\gamma_{s}^{tot.}$, followed by alumina particles and monogal-Zn surfaces, whereas, the oxidized carbon fibers and alumina surfaces gave larger values of $\gamma_{s}^{+}$ again, confirming the highest acid–base properties of these materials. The determination of the ratio $\gamma_{s}^{AB}/\gamma_{s}^{d}$ of the solid materials showed that this ratio varies from 12% for ZnO particles to reaching 70% for the oxidized carbon fibers and about 50% for silica and alumina surfaces. This clearly proved the strong contribution of acid–base surface energy relative to the corresponding London dispersive energy.

The application of this new method using the London dispersion equation to several solid surfaces allowed obtaining a net separation between the dispersive and polar free energy of adsorbed polar molecules on solid materials. The various chromatographic methods or models previously used in the literature showed their insufficiencies in giving accurate values of the thermodynamic surface properties of solid particles. The results obtained by different previous IGC methods can be only considered qualitative. Several problems were raised: one of these issues supposed that the surface area and the London dispersive surface energy of organic molecules are constant [4]; another encountered problem was that the methods proposed by Schultz et al. [4], Papirer et al. [3], Donnet et al. [5], Brendlé et al. [6,7], and Chehimi et al. [9] did not arrive to an accurate separation between the dispersive and polar variables of interaction between solid surfaces and polar molecules. We showed in previous studies [28,29,30,31,32] that the surface area of molecules depends on the temperature. The present work showed that the Donnet et al. method [5] cannot be used for an accurate evaluation of the dispersive and polar interactions of materials. The London dispersion equation, theoretically well founded, was applied by taking into consideration the polarizability and ionization energy of probes and solid materials. This allowed for the separation of the polar and dispersive free energy of the interactions by applying this new method to various solid surfaces.

## 3. Methods and Models 

The inverse gas chromatography (IGC) technique [36,37,38,39,40,41,42,43,44] was used in this study to characterize the surface properties of the above solid surfaces. IGC allowed us to obtain the net retention time and, therefore, the net retention volume of the various solvents adsorbed on the different solid materials. This allowed us to obtain the free energy of adsorption $∆G_{a}^{0}$ of the adsorbed molecules by using the following fundamental equation of IGC:

(8) $$∆G_{a}^{0}\left(T\right)=-RTlnVn+C(T)$$

where C(T) is a constant depending on the temperature and the parameters of interaction between the solid and the solvent.

The total free energy of adsorption $∆G_{a}^{0}(T)$ is composed of the respective London dispersive energy $∆G_{a}^{d}(T)$ and polar energy $∆G_{a}^{sp}(T)$:

(9) $$∆G_{a}^{0}\left(T\right)=∆G_{a}^{d}(T)+∆G_{a}^{sp}(T)$$

To better quantify the polar contribution of the interaction between solid materials and organic molecules, we used an original method based on the expression of the London dispersion interaction. In the next section, we gave the theoretical development of this interesting equation.

### 3.1. London Dispersion Interaction Energy [33]

**Figure 4:**
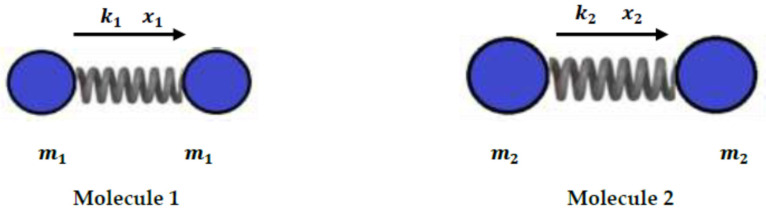
Two non-polar molecules in interactions with mutual fluctuations.

Let us consider two non-polar molecules, 1 and 2, of respective masses $m_{1}$ and $m_{2}$ with an induced dipole–induced dipole interaction. Molecules 1 and 2 can be then represented by two uncoupled oscillators of respective stiffness constants $k_{1}$ and $k_{2}$. The resulting mutual fluctuations are given by the displacements $x_{1}$ and $x_{2}$ of Molecules 1 and 2 at equilibrium (Figure 4).

The respective potential energies of these fluctuations are given by Relation (10):

(10) $$\left\{\begin{array}{c} u_{1}=\frac{1}{2}k_{1}x_{1}^{2}=m_{1}\omega_{1}^{2}x_{1}^{2}\\ u_{2}=\frac{1}{2}k_{2}x_{2}^{2}=m_{2}\omega_{2}^{2}x_{2}^{2} \end{array}\right.$$

where $\omega_{1}$ and $\omega_{2}$., the respective pulsations of the above oscillators, are expressed by Relation (11):

(11) $$\left\{\begin{array}{c} \omega_{1}=\sqrt{\frac{k_{1}}{m_{1}}}\\ \omega_{2}=\sqrt{\frac{k_{2}}{m_{2}}} \end{array}\right.$$

The potential energy $u_{p}$ of fluctuations can be written as:

(12) $$u_{p}=u_{1}+u_{2}=m_{1}\omega_{1}^{2}x_{1}^{2}+m_{2}\omega_{2}^{2}x_{2}^{2}$$

In order to facilitate the calculations, one supposes that the two molecules, 1 and 2, are identical and each oscillator exhibits a charge q. Therefore, the induced dipole moments $\mu_{1}$ and $\mu_{2}$ can be written as:

(13) $$\left\{\begin{array}{c} \mu_{1}=qx_{1}\\ \mu_{2}=qx_{2} \end{array}\right.$$

The resulting electrostatic interaction potential $u_{el}\left(x\right)$ of the fluctuating molecules at un equilibrium distance x is then given by Equation (14):

(14) $$u_{el}\left(x\right)=\frac{1}{4\pi \varepsilon_{0}}\left[\frac{q^{2}}{x}-\frac{q^{2}}{x-x_{1}}-\frac{q^{2}}{x+x_{2}}+\frac{q^{2}}{\left(x-x_{1}\right)+x_{2}}\right]$$

By supposing that $x\gg Max(x_{1},x_{2})$, putting $\varepsilon_{1x}=\frac{x_{1}}{x}$, $\varepsilon_{2x}=\frac{x_{2}}{x}$, $\varepsilon_{3x}=\frac{x_{2}-x_{1}}{x}$ and using the series expansion, on writes:

(15) $$u_{el}\left(x\right)=\frac{1}{4\pi \varepsilon_{0}}\frac{q^{2}}{x}\left[1-{\left(1-\varepsilon_{1x}\right)}^{-1}-{\left(1+\varepsilon_{2x}\right)}^{-1}+{\left(1+\varepsilon_{3x}\right)}^{-1}\right]$$

Using the following series expansions:

$$\left\{\begin{array}{c} {\left(1-\varepsilon_{1x}\right)}^{-1}=1+\sum_{m=1}^{\infty }{\varepsilon_{1x}}^{m}\;\;\;\;\;\;\;\;\;\;\;\;\\ {\left(1+\varepsilon_{2x}\right)}^{-1}=1+\sum_{m=1}^{\infty }{\left(-1\right)}^{m}{\varepsilon_{2x}}^{m}\\ {\left(1+\varepsilon_{3x}\right)}^{-1}=1+\sum_{i=1}^{\infty }{{{\left(-1\right)}^{m}\varepsilon }_{3x}}^{m} \end{array}\right.$$

The electrostatic interaction energy can be written as:

(16) $$u_{el}\left(x\right)=\frac{1}{4\pi \varepsilon_{0}}\frac{q^{2}}{x}\sum_{m=1}^{\infty }\left[-{\varepsilon_{1x}}^{m}-{\left(-1\right)}^{m}{\varepsilon_{2x}}^{m}+{{{\left(-1\right)}^{m}\varepsilon }_{3x}}^{m}\right]$$

By proving that the terms of the first order (in $\frac{1}{x}$) are canceled and by limiting the series expansion to the third order, the expression of the electrostatic interaction energy becomes:

(17) $$u_{el}\left(x\right)=-\frac{q^{2}}{2\pi \varepsilon_{0}}\frac{x_{1}x_{2}}{x^{3}}$$

The total energy of the system (Equation (18)) is composed of the sum of the oscillator interaction energy and the electrostatic interaction energy:

(18) $$u_{total}(x)=u_{p}+u_{el}\left(x\right)$$

Now, by supposing that the two molecules are identical (and then identical oscillators), with a mass m, k the spring stiffness constant of the oscillator, and $\varepsilon_{0}$ its pulsation ($\omega_{0}=\sqrt{\frac{k}{m}}$), the total interaction energy can be written as:

(19) $$u_{total}\left(x\right)=\frac{1}{2}kx_{1}^{2}+\frac{1}{2}kx_{1}^{2}-\frac{q^{2}}{2\pi \varepsilon_{0}}\frac{x_{1}x_{2}}{x^{3}}$$

This equation can be easily transformed to the following form (Equation 20):

(20) $$u_{total}\left(x\right)=\frac{1}{2}\left(k-\frac{q^{2}}{2\pi \varepsilon_{0}x^{3}}\right){\left(\frac{x_{1}+x_{2}}{\sqrt{2}}\right)}^{2}+\frac{1}{2}\left(k+\frac{q^{2}}{2\pi \varepsilon_{0}x^{3}}\right){\left(\frac{x_{1}-x_{2}}{\sqrt{2}}\right)}^{2}$$

The electrostatic potential energy in the expression of the total energy of the interaction will affect the vibrational frequency of each spring. Two new equivalent spring stiffness constants $k_{L}$ and $k_{M}$ are proposed when Schrödinger’s equation is used to describe the system. Two new equivalent displacements $x_{L}$ and $x_{M}$ are also deduced (Equation (21)):

(21) $$\left\{\begin{array}{c} k_{L}=k-\frac{q^{2}}{2\pi \varepsilon_{0}x^{3}}\\ k_{M}=k+\frac{q^{2}}{2\pi \varepsilon_{0}x^{3}}\\ x_{L}=\frac{x_{1}+x_{2}}{\sqrt{2}}\;\;\;\;\;\;\;\;\\ x_{M}=\frac{x_{1}-x_{2}}{\sqrt{2}}\;\;\;\;\;\;\; \end{array}\right.$$

In such a way that the total interaction energy can be written as:

(22) $$u_{total}\left(x\right)=\frac{1}{2}k_{L}{x_{L}}^{2}+\frac{1}{2}k_{M}{x_{M}}^{2}$$

The equivalent new pulsations, $\omega_{L}$ and $\omega_{M}$, of the system can be therefore given by:

(23) $$\left\{\begin{array}{c} \omega_{L}=\sqrt{\frac{k_{L}}{m}}=\sqrt{\frac{k-\frac{q^{2}}{2\pi \varepsilon_{0}x^{3}}}{m}}\\ \omega_{M}=\sqrt{\frac{k_{M}}{m}}=\sqrt{\frac{k+\frac{q^{2}}{2\pi \varepsilon_{0}x^{3}}}{m}} \end{array}\right.$$

The interaction energy change ∆u for the uncoupled system can be given by Equation (24):

(24) $$∆u\left(x\right)=\frac{1}{2}\left(\frac{h\omega_{L}}{2\pi }+\frac{h\omega_{M}}{2\pi }\right)-2\times \frac{1}{2}\frac{h\omega_{0}}{2\pi }$$

Or

(25) $$∆u\left(x\right)=\frac{h}{4\pi }\left(\omega_{L}+\omega_{M}-2\omega_{0}\right)$$

By putting

$$\varepsilon_{x}=\frac{q^{2}}{2\pi \varepsilon_{0}kx^{3}}\left(\varepsilon \ll 1\right)$$

$∆u\left(x\right)$ can be given by Equation (26):

(26) $$∆u\left(x\right)=\frac{h}{4\pi }\sqrt{\frac{k}{m}}\left[{\left(1-\varepsilon_{x}\right)}^{1/2}+{\left(1+\varepsilon_{x}\right)}^{1/2}-2\right]$$

Using the following limited developments until Order 3:

$${\left(1-\varepsilon_{x}\right)}^{1/2}=1-\frac{1}{2}\varepsilon_{x}-\frac{1}{8}{\varepsilon \left(x\right)}^{2}-\frac{1}{16}{\varepsilon_{x}}^{3}+o({\varepsilon_{x}}^{3})$$

$${\left(1+\varepsilon_{x}\right)}^{1/2}=1+\frac{1}{2}\varepsilon_{x}-\frac{1}{8}{\varepsilon_{x}}^{2}+\frac{1}{16}{\varepsilon_{x}}^{3}+o({\varepsilon_{x}}^{3})$$

$∆u\left(x\right)$ can be written as:

(27) $$∆u\left(x\right)=\frac{h}{4\pi }\sqrt{\frac{k}{m}}\;\left[-\frac{1}{4}{\varepsilon_{x}}^{2}\right]$$

By using the expression of ε(x) and $\omega_{0}=\sqrt{\frac{k}{m}}$, the interaction energy variation can be obtained in a one-dimensional system $\left(x\right)$:

(28) $$∆u\left(x\right)=-\frac{1}{2}\frac{h\omega_{0}}{2\pi }\;{\left(\frac{q^{2}}{4\pi \varepsilon_{0}k}\right)}^{2}\frac{1}{x^{6}}$$

It is well known that the electrostatic force qE at equilibrium is balanced by the return force $kx_{2}$ of the spring:

(29) $$qE=kx_{2}$$

where E is the created electric field that induces a dipole moment $\mu_{induced}$ given by:

(30) $$\mu_{induced}=qx_{2}=\frac{q^{2}}{k}E=\alpha_{0}E$$

The polarizability α is expressed as:

(31) $$\alpha_{0}=\frac{q^{2}}{k}$$

Therefore, $∆u\left(x\right)$ for similar molecules can be then written as:

(32) $$∆u\left(x\right)=-\frac{1}{2}\frac{h\omega_{0}}{2\pi }\;{\left(\frac{\alpha_{0}}{4\pi \varepsilon_{0}}\right)}^{2}\frac{1}{x^{6}}$$

Equation (32) was obtained for a one-dimensional system. It can be written in a three-dimensional case of two similar molecules separated by a distance H by the corresponding London dispersion interaction energy $∆U_{L}^{d}\left(x\right)$:

(33) $$∆U_{L}^{d}\left(H\right)=-\frac{3}{2}\frac{h\omega_{0}}{2\pi }\;{\left(\frac{\alpha_{0}}{4\pi \varepsilon_{0}}\right)}^{2}\frac{1}{H^{6}}$$

Or

(34) $$∆U_{L}^{d}\left(H\right)=-\frac{3}{2}h\nu_{0}\;{\left(\frac{\alpha_{0}}{4\pi \varepsilon_{0}}\right)}^{2}\frac{1}{H^{6}}$$

where $\nu_{0}$ is the eigenfrequency of the considered molecule.

If ε is the ionization energy of the above molecule, the London equation, Equation (34), relative to identical molecules, becomes [33]:

(35) $$∆U_{L}^{d}\left(H\right)=-\frac{3}{2}\frac{{\alpha_{0}}^{2}\;\varepsilon }{{\left(4\pi \varepsilon_{0}\right)}^{2}}\frac{1}{x^{6}}$$

In the case of non-identical molecules, Equation (36) can be applied for one mole of molecules:

(36) $$∆U_{L}^{d}\left(H\right)=-\frac{3}{2}\frac{\alpha_{01}\;\alpha_{02}}{{\left(4\pi \varepsilon_{0}\right)}^{2}\;H^{6}}\frac{{R\;\nu }_{1}\;\nu_{2}}{\left(\nu_{1}+\nu_{2}\right)}=-\frac{3}{2}\frac{\alpha_{01}\;\alpha_{02}}{{\left(4\pi \varepsilon_{0}\right)}^{2}\;H^{6}}\frac{{N\varepsilon }_{1}\;\varepsilon_{2}}{\left(\varepsilon_{1}+\varepsilon_{2}\right)}$$

where $\alpha_{01}\;$and $\alpha_{02}$ are the respective deformation polarizabilities of Molecules 1 and 2 separated by a distance H, $\varepsilon_{1}$ and $\varepsilon_{2}$ are the ionization energies of Molecules 1 and 2, and $\nu_{1}$ and $\nu_{2}$ are their characteristic electronic frequencies.

### 3.2. London Free Dispersion Energy in IGC at Infinite Dilution

The free dispersive energy $-∆G_{a}^{d}\left(T\right)$ between two non-identical materials was used in inverse gas chromatography to characterize the dispersive interactions of organic molecules adsorbed on solid surfaces. The London dispersion equation [33] given by Relation (36) can here be advantageously applied and one can write the fundamental equation:

(37) $$∆G_{a}^{d}\left(T\right)=∆U_{L}^{d}\left(H\right)=-\frac{3}{2}\frac{\alpha_{01}\;\alpha_{02}}{{\left(4\pi \varepsilon_{0}\right)}^{2}\;H^{6}}\frac{{N\varepsilon }_{1}\;\varepsilon_{2}}{\left(\varepsilon_{1}+\varepsilon_{2}\right)}$$

By denoting S the solid molecule (Molecule 1) and X the probe molecule (Molecule 2) and combining the previous equations, Equations (1)–(3), one obtained Equation (38):

(38) $$∆G_{a}^{0}\left(T\right)=-RTlnVn+C\left(T\right)=-\frac{\alpha_{0S}\;}{\;H^{6}}\left[\frac{3N}{{2\left(4\pi \varepsilon_{0}\right)}^{2}}\left(\frac{\varepsilon_{S}\;\varepsilon_{X}}{\left(\varepsilon_{S}+\varepsilon_{X}\right)}\alpha_{0X}\right)\right]+∆G_{a}^{sp}(T)$$

The thermodynamic parameter $P_{SX}$ chosen as new indicator variable in this original contribution is given by Relation (39):

(39) $$P_{SX}=\frac{\varepsilon_{S}\;\varepsilon_{X}}{\left(\varepsilon_{S}+\varepsilon_{X}\right)}\alpha_{0X}$$

Indeed, the London dispersion interactions strongly depend on the deformation polarizability of the organic molecules and on the ionization energies of the solid and the solvents because the approximation $\frac{\varepsilon_{S}\;\varepsilon_{X}}{\left(\varepsilon_{S}\;+\;\varepsilon_{X}\right)}\approx \frac{\sqrt{{\varepsilon_{S}\;\varepsilon }_{X}}}{2}$ is not always valid and depends on the product of the ionization energies $\varepsilon_{S}\;\varepsilon_{X}$. To avoid any source of errors on the determination of the London dispersive and polar energies, one chose to use the true values of the ionization energies and not the approximation of the geometric mean.

Now, by drawing the variations of the RTlnVn of n-alkanes adsorbed on the solid material as a function of $\left[\frac{3N}{{2\left(4\pi \varepsilon_{0}\right)}^{2}}\left(\frac{\varepsilon_{S}\;\varepsilon_{X}}{\left(\varepsilon_{S}\;+\;\varepsilon_{X}\right)}\alpha_{0X}\right)\right]$ at a fixed temperature *T*, one obtained the linear equation given by (40):

(40) $$RTlnVn\left(n-alkane\right)=A\left[\frac{3N}{{2\left(4\pi \varepsilon_{0}\right)}^{2}}P_{SX\left(n-alkane\right)}\right]-C$$

where A is the slope of the n-alkanes straight line given by (41):

(41) $$A=\frac{\alpha_{0S}\;}{\;H^{6}}$$

**Figure 5:**
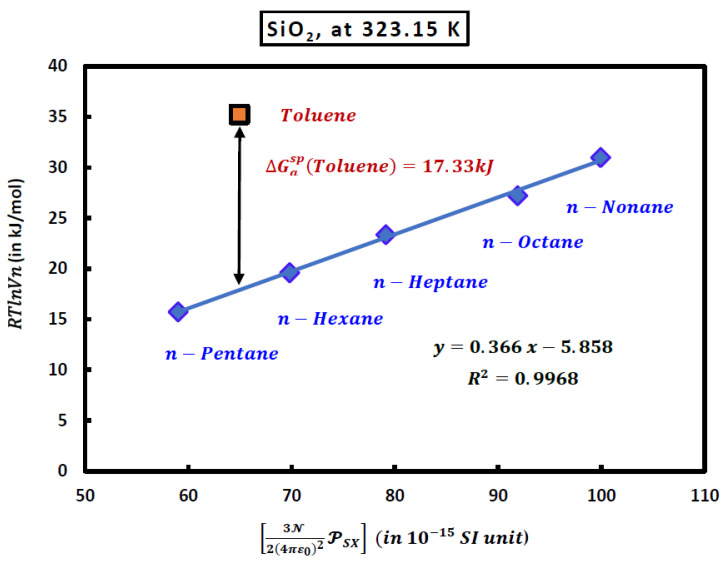
Variations of the RTlnVn of n-alkanes and toluene adsorbed on the silica particles as a function of $\left[\frac{3N}{{2\left(4\pi \varepsilon_{0}\right)}^{2}}P_{SX}\right]$ at *T* = 323.15 K.

In the case of an adsorbed polar organic molecule, such as toluene, the distance between its representative point given by $RTlnVn\left(Toluene\right)$ and the straight line of n-alkanes shown in Figure 5 allowed us to obtain the polar free energy $∆G_{a}^{sp}\left(Toluene\right)$ (London dispersion interaction).

The numerical value of the London dispersion interaction of toluene (in kJ/mol) adsorbed on silica particles is given by the following equation:

(42) $$∆G_{a}^{sp}=RTlnVn-\left[0.366(\mathrm{in}\;\mathrm{kJ}/(10^{-15}\;\mathrm{SI}))\right]\times \frac{3N}{{2\left(4\pi \varepsilon_{0}\right)}^{2}}P_{SiO_{2}-Toluene}\;(\mathrm{in}\;10^{-15}\;\mathrm{SI})$$

Experimental results were given at 323.15 K:

(43) $$RTlnVn\left(Toluene\right)=35.225\;\mathrm{kJ}/\mathrm{mol};\;\frac{3N}{{2\left(4\pi \varepsilon_{0}\right)}^{2}}P_{SiO_{2}-Toluene}=64.954\times 10^{-15}\;\mathrm{SI}\;\mathrm{unit}$$

At this temperature, one obtained the value of the specific free energy of toluene from (43):

(44) $$∆G_{a}^{sp}\left(Toluene\right)=17.330\;\mathrm{kJ}/\mathrm{mol}$$

By varying the temperature, the calculations allowed us to determine the variations of the $∆G_{a}^{sp}\left(T\right)$ of polar probes as a function of the temperature and obtain the specific enthalpy $\left(-∆H_{a}^{sp}\right)$ and entropy $\left(∆S_{a}^{sp}\right)$ of the various polar probes adsorbed on the solid surfaces from Equation (45a):

(45a) $$∆G_{a}^{sp}\left(T\right)=∆H_{a}^{sp}\;-\;∆S_{a}^{sp}$$

If the linearity of $∆G_{a}^{sp}\left(T\right)$ is not verified, the following relations can be used to deduce the variations of the polar enthalpy and entropy of the adsorbed molecules as a function of the temperature:

(45b) $$\left\{\begin{array}{c} ∆H_{a}^{sp}(T)=\frac{𝜕\left(\frac{∆G_{a}^{sp}\left(T\right)}{T}\right)}{𝜕\left(\frac{1}{T}\right)}\\ ∆S_{a}^{sp}(T)=-\left(\frac{𝜕\left(∆G_{a}^{sp}\left(T\right)\right)}{𝜕T}\right) \end{array}\right.$$

This will allow the deduction of the Lewis acid–base constants *K_A_* and *K_D_* by Equation (46):

(46) $$-∆H^{Sp}=\;K_{A}\times DN+K_{D}\times AN$$

where *AN* and *DN* are, respectively, the electron donor and acceptor numbers of the polar molecule. These numbers were calculated by Gutmann [45] and corrected by Fowkes [46].

This was achieved by using the representation $\frac{-∆H^{Sp}}{AN}=f\left(\frac{DN}{AN}\right)$ and Equation (47):

(47) $$-\frac{∆H^{Sp}}{AN}=K_{A}\;\frac{DN}{AN}+K_{D}$$

The slope of the straight line gave the acidic constant $K_{A}\;$whereas the basic constant $K_{D}$ is obtained by the ordinate at the origin of the straight line given by Equation (47).

However, in many cases, one proved that Equation (13) is not verified and one previously proposed another relation taking into account the amphoteric effect of the solid material [47]:

(48) $$-∆H^{Sp}=\;K_{A}\times DN+K_{D}\times AN-K_{CC}\times AN\times DN$$

where $K_{CC}$ is the coupling constant representing the amphoteric character of the material.

Equation (48) can be written as:

(49) $$-\frac{∆H^{Sp}}{AN}=K_{A}\;\frac{DN}{AN}+K_{D}-K_{CC}\times DN$$

By considering a polar molecule symbolized by $\left(i\right)$ and putting:

(50) $$\left\{\begin{array}{c} {\left(x_{1}\right)}_{i}=-\frac{∆H^{Sp}}{AN}\\ {\left(x_{2}\right)}_{i}=\;\;\;\;\frac{DN}{AN}\;\;\\ {\left(x_{3}\right)}_{i}={\;\;\;\;\;K}_{D}\;\;\; \end{array}\right.$$

One can write the general equation, Equation (51), representing any polar molecule $\left(i\right)$ in interaction with solid surfaces:

(51) $${\left(x_{1}\right)}_{i}=K_{D}+K_{A}\;{\left(x_{2}\right)}_{i}-K_{CC}\times {\left(x_{3}\right)}_{i}$$

where ${\left(x_{1}\right)}_{i}$, ${\left(x_{2}\right)}_{i}$, and ${\left(x_{3}\right)}_{i}$ are experimentally well known whereas $K_{D}$, $K_{A}$, and $K_{CC}$ are the unknown quantities of the problem (51).

For n-polar molecules (n≥3), the solution of the linear system (51) can be obtained by the least squares method by finding the vector $\left(K_{D};\;K_{A};-K_{CC}\right)$ that minimizes the sum of the squares of the residuals. 

In this case, the system of Equation (51) will be transformed into a linear system represented by the following equations:

(52) $$\left\{\begin{array}{c} \sum_{i=1}^{n}{\left(x_{1}\right)}_{i}=K_{D}n+K_{A}\;\sum_{i=1}^{n}{\left(x_{2}\right)}_{i}-K_{CC}\sum_{i=1}^{n}{\left(x_{3}\right)}_{i}\;\;\;\;\;\;\;\;\;\;\;\;\;\;\;\;\;\;\;\;\;\;\;\;\;\;\;\;\;\;\;\;\;\;\;\;\;\;\;\;\;\;\;\;\;\;\;\;\\ \sum_{i=1}^{n}\left[{\left(x_{1}\right)}_{i}{\left(x_{2}\right)}_{i}\right]=K_{D}\sum_{i=1}^{n}{\left(x_{2}\right)}_{i}+K_{A}\;\sum_{i=1}^{n}{\left[{\left(x_{2}\right)}_{i}\right]}^{2}-K_{CC}\sum_{i=1}^{n}\left[{\left(x_{2}\right)}_{i}{\left(x_{3}\right)}_{i}\right]\;\;\\ \sum_{i=1}^{n}\left[{\left(x_{1}\right)}_{i}{\left(x_{3}\right)}_{i}\right]=K_{D}\sum_{i=1}^{n}{\left(x_{3}\right)}_{i}+K_{A}\;\sum_{i=1}^{n}\left[{\left(x_{2}\right)}_{i}{\left(x_{3}\right)}_{i}\right]-K_{CC}\sum_{i=1}^{n}{\left[{\left(x_{3}\right)}_{i}\right]}^{2} \end{array}\right.$$

Equation (18) can be represented by the following matrix system:

(53) $$\left(\begin{array}{ccc} n\;\;\;\;\;\;\;\;\; & \sum_{i=1}^{n}{\left(x_{2}\right)}_{i}\;\;\;\;\;\;\;\;\;\; & \sum_{i=1}^{n}{\left(x_{3}\right)}_{i}\;\;\;\;\;\;\;\;\;\;\;\;\\ \sum_{i=1}^{n}{\left(x_{2}\right)}_{i} & \sum_{i=1}^{n}{\left[{\left(x_{2}\right)}_{i}\right]}^{2}\;\;\;\; & \sum_{i=1}^{n}\left[{\left(x_{2}\right)}_{i}{\left(x_{3}\right)}_{i}\right]\\ \sum_{i=1}^{n}{\left(x_{3}\right)}_{i} & \sum_{i=1}^{n}\left[{\left(x_{2}\right)}_{i}{\left(x_{3}\right)}_{i}\right] & \sum_{i=1}^{n}{\left[{\left(x_{3}\right)}_{i}\right]}^{2}\;\;\;\;\;\;\; \end{array}\right)\times \left(\begin{array}{c} K_{D}\\ K_{A}\\ -K_{CC} \end{array}\right)=\left(\begin{array}{c} \sum_{i=1}^{n}{\left(x_{1}\right)}_{i}\;\;\;\;\;\;\;\;\;\;\;\;\\ \sum_{i=1}^{n}\left[{\left(x_{1}\right)}_{i}{\left(x_{2}\right)}_{i}\right]\\ \sum_{i=1}^{n}\left[{\left(x_{1}\right)}_{i}{\left(x_{3}\right)}_{i}\right] \end{array}\right)$$

Symbolized by the matrix equation:

(54) AX=B

The matrix equation is inversible because Matrix A is symmetric and then there is a unique solution $X=\left(K_{D};\;K_{A};-K_{CC}\right)$ given by the formal Equation (55):

(55) $$X=A^{-1}\times B$$

Our method was used in all solid materials that did not satisfy the classic equation, Equation (47).

In this study, one also determined the Lewis entropic acidic $\omega_{A}$ and basic $\omega_{D}$ parameters to obtain the Lewis entropic acid–base character of the solid materials. Equations (56) and (57) were given by analogy of that of the Lewis enthalpic acid–base constants $K_{A}$ and $K_{D}$:

(56) $$\left(-∆S_{a}^{sp}\right)=\omega_{A}\;{DN}^{\prime }+\omega_{D}\;{AN}^{\prime }$$

or

(57) $$\left(\frac{-∆S_{a}^{sp}}{{AN}^{\prime }}\right)=\omega_{A}\;\left(\frac{{DN}^{\prime }}{{AN}^{\prime }}\right)+\omega_{D}$$

## 4. Materials and Solvents

One used, in this paper, several solid materials, such as silica (SiO_2_), alumina (Al_2_O_3_), magnesium oxide (MgO), zinc oxide (ZnO), Monogal-Zn, titanium dioxide (TiO_2_), and carbon fibers that were characterized in previous papers [28,29,30,31,32] with other chromatographic methods and molecular models. The organic solvents, such as n-alkanes and polar molecules, were those previously used in other studies. The donor and acceptor numbers of electrons used in this paper were those calculated and corrected by Riddle and Fowkes [46]. The chromatographic measurements were obtained from a Focus GC Chromatograph equipped with a flame ionization detector of high sensitivity. All experimental methods of this technique were previously explained in detail in previous papers [28,29,30,31,32].

## 5. Conclusions

A new and original method of the separation of London dispersive and polar surface energy was proposed by using the inverse gas chromatography (IGC) technique at infinite dilution. This method used the London dispersion interaction equation. A theoretical demonstration was developed by taking into account the polarizability and ionization energy of studied solids and adsorbed molecules. The parameter of the polarizability of organic molecules adsorbed on eight different solid materials was used to propose a new parameter taking into account all terms involved in the expression of the London dispersive energy of interactions. The originality of this new method concerned the full determination and use of a new intrinsic thermodynamic parameter $P_{SX}=\frac{\varepsilon_{S}\;\varepsilon_{X}}{\left(\varepsilon_{S}\;+\;\varepsilon_{X}\right)}\alpha_{0X}$ reflecting the London dispersive energy of the interaction between solid materials and organic molecules. One calculated the parameter $P_{SX}$ for different materials and organic molecules. Experimental results obtained by IGC allowed us to determine the average separation distance of solid-organic solvents at different temperatures. The dispersive free energy and the polar energy of n-alkanes and polar probes were determined by this method. The thermal model was used to quantify the London dispersive surface energy $\gamma_{s}^{d}(T)$ of the various solid materials at different temperatures and allowed us to determine the different components $\gamma_{s}^{+}$, $\gamma_{s}^{-}$, and $\gamma_{s}^{AB}$ of acid–base surface energies of solid particles, as well as their total surface energy $\gamma_{s}^{tot.}$. Results showed the highest acid–base surface energy was obtained by the oxidized carbon fibers followed by silica particles and alumina surfaces.

The determination of the polar interaction energy $∆G_{a}^{sp}\left(T\right)$ of the different polar molecules adsorbed on the solid materials allowed us to obtain the polar enthalpy and entropy of the interaction and, therefore, the enthalpic and entropic Lewis acid–base constants. The results showed that all studied solid surfaces exhibited amphoteric behavior with stronger Lewis’s basicity. The oxidized and untreated carbon fibers, ZnO, and silica particles showed an important basic force whereas silica, alumina, and monogal-Zn presented the highest Lewis’s acidity.

The application of the classic equation allowing the determination of the acid–base constants showed poor linear regression coefficients. It was corrected by using the Hamieh model that added a coupling constant reflecting the amphoteric character of solid materials.

It was proved that the method proposed by Donnet et al. neglected the values of the harmonic mean $\bar{\varepsilon_{S-X}}$ of the ionization energies of solids and solvents and this resulted in a surestimation of the specific or polar free energy of the interaction reaching, in several cases, five times the corrected value. By taking into account the different values of harmonic mean and the deformation polarizability of n-alkanes and polar organic molecules, one obtained more accurate values of the London dispersive energy, the polar energy, the acid–base constants, and the acid–base surface energies of the various solids in interaction with several polar molecules.

The different theoretical and experimental results obtained by this work can be very useful in the different industrial processes of adhesion, catalysis, pharmaceutics, and biomaterials where the dispersive, polar surface energy and Lewis’s acid–base properties play an important role in the selection criteria of the best solid materials exhibiting the best physicochemical surface properties.

## Figures and Tables

**Table 1:** Values of deformation polarizability $\alpha_{0}$ (respectively in 10^−30^ m^3^ and in 10^−40^ C m^2^/V) and ionization energy ε (in eV) of the various organic molecules and solid materials.

Molecule	$\varepsilon_{X}$$\;\mathrm{or}\;\varepsilon_{S}$ (eV)	$\alpha_{0X}$$\;\mathrm{or}\;\alpha_{0S}$ (in 10^−30^ m^3^)	$\alpha_{0X}$$\;\mathrm{or}\;\alpha_{0S}$ (in 10^−40^ C m^2^/V)
n-pentane	10.28	9.99	11.12
n-hexane	10.13	11.90	13.24
n-heptane	9.93	13.61	15.14
n-octane	9.80	15.90	17.69
n-nonane	9.71	17.36	19.32
n-decane	9.65	19.10	21.25
CCl_4_	11.47	10.85	12.07
Nitromethane	11.08	7.37	8.20
CH_2_Cl_2_	11.32	7.21	8.02
CHCl_3_	11.37	8.87	9.86
Diethyl ether	9.51	9.47	10.54
Tetrahydrofuran	9.38	8.22	9.15
Ethyl acetate	10.01	9.16	10.19
Acetone	9.70	6.37	7.09
Acetonitrile	12.20	4.44	4.94
Toluene	8.83	11.80	13.13
Benzene	9.24	10.35	11.52
Methanol	10.85	3.28	3.65
SiO_2_	8.15	5.42	6.04
MgO	7.65	5.47	6.09
ZnO	4.35	5.27	5.86
Zn	9.39	5.82	6.47
Al_2_O_3_	5.99	5.36	5.96
TiO_2_	9.50	7.12	7.92
Carbon	11.26	1.76	1.96

**Table 2:** Values of the harmonic mean of the ionization energies of SiO_2_ particles and organic solvents (in 10^−19^ J) and the parameter $\frac{3N}{{2\left(4\pi \varepsilon_{0}\right)}^{2}}P_{SiO_{2}-X}$ (in 10^−15^ SI unit) for the various organic molecules.

Molecule	$\frac{\varepsilon_{SiO_{2}}\varepsilon_{X}}{\left(\varepsilon_{SiO_{2}}\;+\;\varepsilon_{X}\right)}$ (in 10^−19^ J)	$\frac{3N}{{2\left(4\pi \varepsilon_{0}\right)}^{2}}P_{SiO_{2}-X}$ (in 10^−15^ SI)
n-pentane	7.274	58.992
n-hexane	7.226	69.814
n-heptane	7.162	79.135
n-octane	7.119	91.901
n-nonane	7.089	99.919
n-decane	7.069	109.623
CCl_4_	7.623	67.151
Nitromethane	7.513	44.956
CH_2_Cl_2_	7.582	44.379
CHCl_3_	7.596	54.666
Diethyl ether	7.022	53.988
Tetrahydrofuran	6.977	46.564
Ethyl acetate	7.188	53.453
Acetone	7.087	36.652
Acetonitrile	7.818	28.180
Toluene	6.780	64.955
Benzene	6.930	58.231
Methanol	7.447	19.829

**Table 3:** Values of the harmonic mean of the ionization energies of MgO particles and organic solvents (in 10^−19^ J) and the parameter $\frac{3N}{{2\left(4\pi \varepsilon_{0}\right)}^{2}}P_{MgO-X}$ (in 10^−15^ SI unit) for the various organic molecules.

Molecule	$\frac{\varepsilon_{MgO}\varepsilon_{X}}{\left(\varepsilon_{MgO}\;+\;\varepsilon_{X}\right)}$ (in 10^−19^ J)	$\frac{3N}{{2\left(4\pi \varepsilon_{0}\right)}^{2}}P_{MgO-X}$ (in 10^−15^ SI)
n-pentane	7.018	56.917
n-hexane	6.974	67.374
n-heptane	6.914	76.393
n-octane	6.874	88.735
n-nonane	6.846	96.490
n-decane	6.828	105.872
CCl_4_	7.343	64.680
Nitromethane	7.241	43.325
CH_2_Cl_2_	7.304	42.754
CHCl_3_	7.317	52.662
Diethyl ether	6.783	52.153
Tetrahydrofuran	6.742	44.991
Ethyl acetate	6.938	51.595
Acetone	6.844	35.395
Acetonitrile	7.523	27.117
Toluene	6.557	62.820
Benzene	6.697	56.277
Methanol	7.179	19.116

**Table 4:** Values of the harmonic mean of the ionization energies of ZnO particles and organic solvents (in 10^−19^ J) and the parameter $\frac{3N}{{2\left(4\pi \varepsilon_{0}\right)}^{2}}P_{ZnO-X}$ (in 10^−15^ SI unit) for the various organic molecules.

Molecule	$\frac{\varepsilon_{ZnO}\varepsilon_{X}}{\left(\varepsilon_{ZnO}\;+\;\varepsilon_{X}\right)}$ (in 10^−19^ J)	$\frac{3N}{{2\left(4\pi \varepsilon_{0}\right)}^{2}}P_{ZnO-X}$ (in 10^−15^ SI)
n-pentane	4.891	39.665
n-hexane	4.869	47.041
n-heptane	4.840	53.478
n-octane	4.820	62.224
n-nonane	4.807	67.745
n-decane	4.797	74.392
CCl_4_	5.046	44.451
Nitromethane	4.998	29.904
CH_2_Cl_2_	5.028	29.431
CHCl_3_	5.034	36.231
Diethyl ether	4.776	36.716
Tetrahydrofuran	4.755	31.732
Ethyl acetate	4.852	36.080
Acetone	4.806	24.852
Acetonitrile	5.131	18.494
Toluene	4.662	44.666
Benzene	4.733	39.769
Methanol	4.968	13.230

**Table 5:** Values of the harmonic mean of the ionization energies of Monogal-Zn and organic solvents (in 10^−19^ J) and the parameter $\frac{3N}{{2\left(4\pi \varepsilon_{0}\right)}^{2}}P_{Zn-X}$ (in 10^−15^ SI unit) for the various organic molecules.

Molecule	$\frac{\varepsilon_{Zn}\varepsilon_{X}}{\left(\varepsilon_{Zn}\;+\;\varepsilon_{X}\right)}$ (in 10^−19^ J)	$\frac{3N}{{2\left(4\pi \varepsilon_{0}\right)}^{2}}P_{Zn-X}$ (in 10^−15^ SI)
n-pentane	7.852	63.683
n-hexane	7.797	75.326
n-heptane	7.722	85.324
n-octane	7.672	99.042
n-nonane	7.638	107.648
n-decane	7.615	118.076
CCl_4_	8.261	72.769
Nitromethane	8.132	48.658
CH_2_Cl_2_	8.212	48.070
CHCl_3_	8.228	59.222
Diethyl ether	7.560	58.122
Tetrahydrofuran	7.508	50.105
Ethyl acetate	7.752	57.650
Acetone	7.635	39.486
Acetonitrile	8.490	30.603
Toluene	7.280	69.743
Benzene	7.453	62.627
Methanol	8.054	21.447

**Table 6:** Values of the harmonic mean of the ionization energies of ${Al}_{2}O_{3}$ and organic solvents (in 10^−19^ J) and the parameter $\frac{3N}{{2\left(4\pi \varepsilon_{0}\right)}^{2}}P_{{Al}_{2}O_{3}-X}$ (in 10^−15^ SI unit) for the various organic molecules.

Molecule	$\frac{\varepsilon_{{Al}_{2}O_{3}}\varepsilon_{X}}{\left(\varepsilon_{{Al}_{2}O_{3}}\;+\;\varepsilon_{X}\right)}$ (in 10^−19^ J)	$\frac{3N}{{2\left(4\pi \varepsilon_{0}\right)}^{2}}P_{{Al}_{2}O_{3}-X}$ (in 10^−15^ SI)
n-pentane	6.056	49.114
n-hexane	6.023	58.186
n-heptane	5.978	66.053
n-octane	5.948	76.784
n-nonane	5.927	83.541
n-decane	5.913	91.697
CCl_4_	6.296	55.460
Nitromethane	6.221	37.222
CH_2_Cl_2_	6.268	36.687
CHCl_3_	6.277	45.177
Diethyl ether	5.880	45.209
Tetrahydrofuran	5.849	39.033
Ethyl acetate	5.996	44.590
Acetone	5.926	30.646
Acetonitrile	6.428	23.171
Toluene	5.710	54.699
Benzene	5.816	48.867
Methanol	6.175	16.443

**Table 7:** Values of the harmonic mean of the ionization energies of $TiO_{2}$ and organic solvents (in 10^−19^ J) and the parameter $\frac{3N}{{2\left(4\pi \varepsilon_{0}\right)}^{2}}P_{TiO_{2}-X}$ (in 10^−15^ SI unit) for the various organic molecules.

Molecule	$\frac{\varepsilon_{TiO_{2}}\varepsilon_{X}}{\left(\varepsilon_{TiO_{2}}\;+\;\varepsilon_{X}\right)}$ (in 10^−19^ J)	$\frac{3N}{{2\left(4\pi \varepsilon_{0}\right)}^{2}}P_{TiO_{2}-X}$ (in 10^−15^ SI)
n-pentane	7.900	64.071
n-hexane	7.844	75.781
n-heptane	7.768	85.834
n-octane	7.718	99.631
n-nonane	7.683	108.285
n-decane	7.660	118.773
CCl_4_	8.314	73.236
Nitromethane	8.183	48.965
CH_2_Cl_2_	8.264	48.376
CHCl_3_	8.281	59.600
Diethyl ether	7.604	58.462
Tetrahydrofuran	7.552	50.396
Ethyl acetate	7.799	57.996
Acetone	7.680	39.719
Acetonitrile	8.546	30.804
Toluene	7.321	70.137
Benzene	7.496	62.988
Methanol	8.104	21.581

**Table 8:** Values of the harmonic mean of the ionization energies of carbon fibers and organic solvents (in 10^−19^ J) and the parameter $\frac{3N}{{2\left(4\pi \varepsilon_{0}\right)}^{2}}P_{Carbon-X}$ (in 10^−15^ SI unit) for the various organic molecules.

Molecule	$\frac{\varepsilon_{Carbon}\varepsilon_{X}}{\left(\varepsilon_{Carbon}\;+\;\varepsilon_{X}\right)}$ (in 10^−19^ J)	$\frac{3N}{{2\left(4\pi \varepsilon_{0}\right)}^{2}}P_{Carbon-X}$ (in 10^−15^ SI)
n-pentane	8.598	69.736
n-hexane	8.532	82.430
n-heptane	8.443	93.286
n-octane	8.384	108.220
n-nonane	8.342	117.574
n-decane	8.314	128.928
CCl_4_	9.091	80.082
Nitromethane	8.935	53.465
CH_2_Cl_2_	9.032	52.869
CHCl_3_	9.052	65.147
Diethyl ether	8.249	63.421
Tetrahydrofuran	8.188	54.640
Ethyl acetate	8.479	63.053
Acetone	8.339	43.125
Acetonitrile	9.369	33.772
Toluene	7.917	75.847
Benzene	8.122	68.249
Methanol	8.841	23.543

**Table 9:** Values of deformation polarizability (in 10^−40^ C m^2^/V) compared to those proposed by Donnet et al. of the various organic molecules, with the calculated relative error.

Molecule	$\alpha_{0X}$$\;\mathrm{or}\;\alpha_{0S}$ (in 10^−40^ C m^2^/V) (Donnet Values)	$\alpha_{0X}$$\;\mathrm{or}\;\alpha_{0S}$ (in 10^−40^ C m^2^/V) (Our Values)	Relative Error (in %)
n-nonane	19.75	19.32	2.2
n-decane	-	21.25	-
CCl_4_	11.68	12.07	3.2
CH_2_Cl_2_	-	8.02	-
CHCl_3_	10.57	9.86	7.2
Diethyl ether	9.71	10.54	8.0
Tetrahydrofuran	8.77	9.15	4.2
Ethyl acetate	10.79	10.19	5.9
Acetone	7.12	7.09	0.4
Acetonitrile	5.43	4.94	10.0
Toluene	13.68	13.13	4.2
Benzene	11.95	11.52	3.7
Methanol	-	3.65	-
SiO_2_	-	6.04	-
MgO	-	6.09	-
ZnO	-	5.86	-
Zn	-	6.47	-
Al_2_O_3_	-	5.96	-
TiO_2_	-	7.92	-
Carbon	-	1.96	-

**Table 10:** Values of the London dispersive surface energy $\gamma_{s}^{d}(T)$ (in mJ/m^2^) of the various solid materials.

Temperature T (K)	323.15	343.15	363.15	383.15	Equation of $\gamma_{s}^{d}\;(T)$
Oxidized carbon fibers	51.59	43.42	35.25	27.08	$\gamma_{s}^{d}\;(T)$ = −0.408 T + 183.6
Untreated carbon fibers	52.96	47.06	41.16	35.27	$\gamma_{s}^{d}\;(T)$ = −0.295 T + 148.2
MgO	54.35	47.92	41.71	35.71	$\gamma_{s}^{d}\;(T)$ = −0.311 T + 154.6
MgO	58.37	53.12	47.87	42.62	$\gamma_{s}^{d}\;(T)$ = −0.262 T + 143.2
ZnO	59.25	55.07	50.12	44.16	$\gamma_{s}^{d}\;(T)$ = −0.251 T + 140.8
Al_2_O_3_	60.98	51.03	41.08	31.13	$\gamma_{s}^{d}\;(T)$ = −0.497 T + 221.7
Monogal-Zn	81.90	68.84	52.26	37.03	$\gamma_{s}^{d}\;(T)$ = −0.756 T + 327.0
SiO_2_	85.34	67.75	52.86	39.23	$\gamma_{s}^{d}\;(T)$ = −0.766 T + 331.8

**Table 11:** Values of $-∆G_{a}^{sp}\left(T\right)$ (in kJ/mol) of the various polar molecules adsorbed on the different used solid materials.

**Silica**				
T(K)	323.15	343.15	363.15	383.15
CCl_4_	6.752	6.810	6.881	6.968
Nitromethane	13.573	12.367	11.273	10.191
CH_2_Cl_2_	22.490	21.846	21.269	20.716
CHCl_3_	19.752	19.304	18.925	18.546
Diethyl ether	26.838	25.462	23.802	22.314
THF	35.506	32.787	30.435	27.908
Ethyl Acetate	4.566	4.015	3.530	3.079
Acetone	10.612	9.608	8.703	7.816
Acetonitrile	16.734	15.304	14.016	12.738
Toluene	17.330	16.724	16.168	15.598
Benzene	5.640	5.170	4.745	4.328
**MgO**				
T(K)	323.1500	343.1500	363.1500	383.15
CH_2_Cl_2_	3.3120	3.7860	4.5320	5.211
CHCl_3_	5.833	2.693	1.560	2.176
Diethyl ether	14.415	16.559	18.671	20.721
THF	23.053	25.004	26.928	28.797
Acetone	15.723	20.520	25.354	30.243
Ethyl acetate	6.224	7.620	9.112	10.523
**ZnO**				
T(K)	323.15	343.15	363.15	383.15
CH_2_Cl_2_	2.4490	1.9151	1.2231	0.6320
CHCl_3_	1.1506	1.0611	0.9988	0.9325
Diethyl ether	7.7211	7.0452	6.5940	6.0373
THF	13.5961	12.9006	12.2948	11.5175
Ethyl acetate	3.9554	2.7149	1.8004	1.0420
Benzene	0.8696	0.6900	0.5367	0.3535
**Monogal-Zn**				
T(K)	323.15	343.15	363.15	383.15
CH_2_Cl_2_	2.354	1.965	1.426	0.854
CHCl_3_	15.001	11.698	7.938	6.927
Diethyl ether	17.481	15.950	14.408	12.982
THF	23.786	21.503	19.298	17.285
Acetone	22.779	20.603	18.500	16.582
Ethyl acetate	12.287	9.154	5.642	4.895
**Alumina**				
T(K)	323.15	343.15	363.15	383.15
CCl_4_	0.334	0.163	0.084	-
CH_2_Cl_2_	6.751	6.654	6.575	6.648
CHCl_3_	38.808	36.648	34.670	32.613
Ether	18.559	16.226	14.028	12.322
THF	41.085	39.144	37.268	35.790
Ethyl acetate	11.624	9.452	7.875	6.125
Toluene	40.532	38.377	36.371	34.878
**TiO_2_**				
T(K)	313.15	333.15	353.15	373.15
CH_2_Cl_2_	2.546	1.924	1.254	0.723
CHCl_3_	3.146	2.019	0.893	-
THF	7.620	6.620	5.620	4.620
Ethyl Acetate	3.979	2.417	0.857	-
Acetone	5.776	4.068	2.362	0.651
Benzene	5.564	4.199	2.834	1.463
Nitromethane	10.394	9.024	7.657	6.283
Acetonitrile	4.615	2.524	0.433	-1.661
**Untreated Carbon fibers**				
T(K)	323.15	343.15	363.15	383.15
CCl_4_	1.723	1.956	2.203	2.518
CH_2_Cl_2_	4.096	3.645	3.129	2.548
CHCl_3_	14.829	13.537	11.761	8.193
Ether	2.112	1.633	1.131	0.546
THF	11.852	11.079	10.310	9.748
C6H6	8.577	8.315	8.055	8.011
Ethyl acetate	9.500	9.251	9.019	8.975
Acetone	10.723	10.282	9.865	9.647
**Oxidized Carbon fibers**				
T(K)	323.15	343.15	363.15	383.15
CCl_4_	2.785	2.843	2.911	2.974
CH_2_Cl_2_	10.546	9.952	9.379	8.800
CHCl_3_	12.788	12.228	11.685	11.134
Ether	7.399	6.965	6.548	6.124
THF	17.020	15.878	14.753	13.623
C6H6	10.429	9.943	9.473	8.995
Ethyl acetate	13.212	12.718	12.242	11.758
Acetone	17.928	16.999	16.094	15.183

**Table 12:** Values of polar enthalpy ($-∆H_{a}^{sp}\;in\;kJ\;{mol}^{-1}$) and entropy ($-∆S_{a}^{sp}\;in\;JK^{-1}\;{mol}^{-1}$) of the various polar solvents adsorbed on the various solid surfaces by using our new method.

Silica		
Polar Solvent	($-∆S_{a}^{sp}\;in\;JK^{-1}\;{mol}^{-1}$)	($-∆H_{a}^{sp}\;in\;kJ\;{mol}^{-1}$)
CCl_4_	−4.6	5.2514
Nitromethane	52.8	30.543
CH_2_Cl_2_	27.7	31.377
CHCl_3_	18.8	25.788
Diethyl ether	77.4	51.914
THF	123.5	75.304
Ethyl acetate	23	11.944
Acetone	43.6	24.624
Acetonitrile	62.2	36.719
Toluene	27.1	26.027
Benzene	20.4	12.173
**MgO**		
CH_2_Cl_2_	32.2	7.1665
CHCl_3_	−60.5	−24.435
Diethyl ether	105.1	19.543
Ethyl acetate	71.9	17.038
THF	95.8	7.8791
Acetone	242	62.489
Acetonitrile	81.6	2.0138
Toluene	−13.8	15.211
**ZnO**		
CH_2_Cl_2_	20.9	8.9949
CHCl_3_	−11.4	1.0743
Diethyl ether	18.5	18.218
THF	23.8	26.647
Ethyl acetate	38.2	17.176
Benzene	−1.0	6.7082
**Monogal**		
CH_2_Cl_2_	25.2	10.547
CHCl_3_	139.9	59.803
Diethyl ether	75.2	41.760
THF	108.5	58.796
Ethyl acetate	44.2	21.674
Acetone	103.5	56.155
Acetonitrile	110.8	54.921
Toluene	99.9	54.474
**Alumina**		
CCl_4_	6.2	2.314
CH_2_Cl_2_	1.9	7.3421
CHCl_3_	102.8	71.989
Diethyl ether	104.6	52.207
THF	88.8	69.683
Ethyl acetate	90.4	40.683
Toluene	94.9	71.036
**Titanium dioxide**		
CH_2_Cl_2_	30.7	12.146
CHCl_3_	56.4	20.818
THF	10.0	23.277
Ethyl Acetate	78.1	28.448
Acetone	85.4	32.518
Benzene	68.3	26.965
Nitromethane	68.5	31.846
Acetonitrile	104.6	37.370
**Untreated carbon fibers**		
CCl_4_	−13.2	−2.4181
CH_2_Cl_2_	25.8	12.209
CHCl_3_	108.4	49.284
Benzene	9.8	11.602
Diethyl ether	26	10.275
THF	35.4	22.895
Ethyl acetate	9	12.289
Acetone	18.2	16.380
**Oxidized carbon fibers**		
CCl_4_	3.2	1.7876
CH_2_Cl_2_	29.1	19.639
CHCl_3_	27.5	21.406
Benzene	23.9	17.897
Diethyl ether	21.2	14.038
THF	56.6	34.733
Ethyl acetate	24.2	20.782
Acetone	45.7	32.230

**Table 13:** Values of the enthalpic acid–base constants $K_{A}$ and $K_{D}\;$(unitless) and the entropic acid base constants $\omega_{A}$ and $\omega_{D}\;$(unitless) of the various solid surfaces and the corresponding acid–base ratios.

Solid Surfaces	$K_{A}$	$K_{D}$	$K_{D}$ $/K_{A}$	$R^{2}$	$10^{3}.\omega_{A}$	$10^{3}.\omega_{D}$	$\omega_{D}$ $/\omega_{A}$	$R^{2}$
Silica	0.73	1.45	2.0	0.6509	1.23	1.45	1.2	0.651
MgO	0.08	1.13	14.0	0.1722	1.16	0.57	0.5	0.8126
ZnO	0.22	1.63	7.4	0.422	0.29	0.08	0.3	0.8761
Monogal-Zn	0.59	1.49	2.5	0.7296	1.07	3.08	2.9	0.7295
Alumina	0.71	2.21	3.1	0.7301	0.92	4.21	4.6	0.7739
Titanium dioxide	0.25	0.87	3.5	0.9874	0.86	1.80	2.1	0.9804
Untreated Carbon fibers	0.13	2.19	16.8	0.0799	0.30	1.56	5.2	0.3195
Oxidized Carbon fibers	0.20	3.41	17.4	0.0779	0.37	4.32	11.6	0.141

**Table 14:** Corrected values of Lewis’s acid–base constants $K_{A}$, $K_{D}\;$and K of the various solid surfaces and the corresponding acid–base ratios.

Solid Surfaces	$K_{A}$	$K_{D}$	K	$K_{D}$ $/K_{A}$
Silica	1.105	3.572	0.186	3.23
MgO	0.005	0.336	−0.045	71.66
ZnO	0.401	2.418	0.089	6.03
Monogal-Zn	0.782	3.477	0.113	4.45
Alumina	0.988	3.291	0.136	3.33
Untreated Carbon fibers	0.359	3.339	0.110	9.29
Oxidized Carbon fibers	0.529	5.085	0.161	9.61

**Table 15:** Values of the ($-∆G_{a}^{sp}\left(T\right)\;in\;kJ\;{mol}^{-1}$), ($-∆S_{a}^{sp}\;in\;JK^{-1}\;{mol}^{-1}$), and ($-∆H_{a}^{sp}\;in\;kJ\;{mol}^{-1}$) of polar molecules adsorbed on silica surfaces by comparing Donnet et al.’s method and our new method.

**Results by Using Donnet et al.’s Method**							
T(K)	323.15	343.15	363.15	383.15	403.15	($-∆S_{a}^{sp}\;in\;JK^{-1}\;{mol}^{-1}$)	($-∆H_{a}^{sp}\;in\;kJ\;{mol}^{-1}$)
CCl_4_	34.616	31.401	28.904	26.818	24.489	124.2	74.643
Nitromethane	34.014	30.151	27.038	24.328	21.424	155	83.687
CH_2_Cl_2_	53.122	48.974	45.622	42.692	39.626	166.4	106.43
CHCl_3_	48.598	44.795	41.775	39.150	36.312	151.1	96.995
Diethyl ether	53.703	49.136	44.982	41.394	37.319	202.5	118.86
THF	57.922	52.382	47.865	43.564	39.053	232.8	132.69
Ethyl Acetate	33.315	29.418	26.298	23.608	20.724	155	82.944
Acetone	25.935	22.701	20.154	18.016	15.527	127.5	66.771
Acetonitrile	25.145	22.059	19.641	17.621	15.228	121.4	64.011
Toluene	55.833	51.069	47.157	43.631	40.161	193.9	117.99
Benzene	38.564	34.399	31.032	28.069	25.006	167.2	92.143
**Results by using our new method**							
T(K)	323.15	343.15	363.15	383.15	403.15	($-∆S_{a}^{sp}\;in\;JK^{-1}\;{mol}^{-1}$)	($-∆H_{a}^{sp}\;in\;kJ\;{mol}^{-1}$)
CCl_4_	6.752	6.810	6.881	6.968	7.129	5.2514	6.752
Nitromethane	13.573	12.367	11.273	10.191	9.378	30.543	13.573
CH_2_Cl_2_	22.490	21.846	21.269	20.716	20.287	31.377	22.490
CHCl_3_	19.752	19.304	18.925	18.546	18.250	25.788	19.752
Diethyl ether	26.838	25.462	23.802	22.314	20.676	51.914	26.838
THF	35.506	32.787	30.435	27.908	25.593	75.304	35.506
Ethyl Acetate	4.566	4.015	3.530	3.079	2.732	11.944	4.566
Acetone	10.612	9.608	8.703	7.816	7.144	24.624	10.612
Acetonitrile	16.734	15.304	14.016	12.738	11.793	36.719	16.734
Toluene	17.330	16.724	16.168	15.598	15.187	26.027	17.330
Benzene	5.640	5.170	4.745	4.328	4.026	12.173	5.640

**Table 16:** Values of the ratios $\frac{\left(-∆G_{a}^{sp}\left(Donnet\;et\;al.\right)\right)}{\left(-∆G_{a}^{sp}\left(Hamieh\right)\right)}$ at different temperatures, $\frac{\left(-∆S_{a}^{sp}\left(Donnet\;et\;al.\right)\right)}{\left(-∆S_{a}^{sp}\left(Hamieh\right)\right)}$, and $\frac{\left(-∆H_{a}^{sp}\left(Donnet\;et\;al.\right)\right)}{\left(-∆H_{a}^{sp}\left(Hamieh\right)\right)}$ of the various polar organic molecules.

T(K)	323.15	343.15	363.15	383.15	403.15	($-∆S_{a}^{sp}\;in\;JK^{-1}\;{mol}^{-1}$)	($-∆H_{a}^{sp}\;in\;kJ\;{mol}^{-1}$)
CCl_4_	5.1	4.6	4.2	3.8	3.4	23.7	11.1
Nitromethane	2.5	2.4	2.4	2.4	2.3	5.1	6.2
CH_2_Cl_2_	2.4	2.2	2.1	2.1	2.0	5.3	4.7
CHCl_3_	2.5	2.3	2.2	2.1	2.0	5.9	4.9
Diethyl ether	2.0	1.9	1.9	1.9	1.8	3.9	4.4
THF	1.6	1.6	1.6	1.6	1.5	3.1	3.7
Ethyl Acetate	7.3	7.3	7.5	7.7	7.6	13.0	18.2
Acetone	2.4	2.4	2.3	2.3	2.2	5.2	6.3
Acetonitrile	1.5	1.4	1.4	1.4	1.3	3.3	3.8
Toluene	3.2	3.1	2.9	2.8	2.6	7.4	6.8
Benzene	6.8	6.7	6.5	6.5	6.2	13.7	16.3

**Table 17:** Harmonic mean $\bar{\varepsilon_{S-X}}$ (in 10^−19^ J) values of the ionization energies of the various materials and the adsorbed polar solvents found in our new approach and values of $\sqrt{\varepsilon_{X}}$ (in 10^−10^ J^1/2^) used by the Donnet et al. method.

Molecule	$\bar{\varepsilon_{SiO2-X}}$ (in 10^−19^ J)	$\bar{\varepsilon_{MgO-X}}$ (in 10^−19^ J)	$\bar{\varepsilon_{ZnO-X}}$ (in 10^−19^ J)	$\bar{\varepsilon_{Zn-X}}$ (in 10^−19^ J)	$\bar{\varepsilon_{Al2O3-X}}$ (in 10^−19^ J)	$\bar{\varepsilon_{TiO2-X}}$ (in 10^−19^ J)	$\bar{\varepsilon_{C-X}}$ (in 10^−19^ J)	$\sqrt{\varepsilon_{X}}$ (in 10^−10^ J^1/2^)
n-pentane	7.27	7.02	4.89	7.85	6.06	7.90	8.60	12.83
n-hexane	7.23	6.97	4.87	7.80	6.02	7.84	8.53	12.73
n-heptane	7.16	6.91	4.84	7.72	5.98	7.77	8.44	12.61
n-octane	7.12	6.87	4.82	7.67	5.95	7.72	8.38	12.52
n-nonane	7.09	6.85	4.81	7.64	5.93	7.68	8.34	12.46
n-decane	7.07	6.83	4.80	7.62	5.91	7.66	8.31	12.43
CCl_4_	7.62	7.34	5.05	8.26	6.30	8.31	9.09	13.55
Nitromethane	7.51	7.24	5.00	8.13	6.22	8.18	8.94	13.32
CH_2_Cl_2_	7.58	7.30	5.03	8.21	6.27	8.26	9.03	13.46
CHCl_3_	7.60	7.32	5.03	8.23	6.28	8.28	9.05	13.49
Diethyl ether	7.02	6.78	4.78	7.56	5.88	7.60	8.25	12.34
Tetrahydrofuran	6.98	6.74	4.76	7.51	5.85	7.55	8.19	12.25
Ethyl acetate	7.19	6.94	4.85	7.75	6.00	7.80	8.48	12.66
Acetone	7.09	6.84	4.81	7.64	5.93	7.68	8.34	12.46
Acetonitrile	7.82	7.52	5.13	8.49	6.43	8.55	8.60	13.97
Toluene	6.78	6.56	4.66	7.28	5.71	7.32	8.53	11.89
Benzene	6.93	7.02	4.73	7.45	5.82	7.50	8.44	12.16
Methanol	7.45	6.97	4.97	8.05	6.18	8.10	8.38	13.18

**Table 18:** Values of the average separation distance H (in Å) between the various solid substrates and the organic molecules at different temperatures.

T(K)	323.15	343.15	363.15	383.15
SiO_2_	5.05	5.12	5.19	5.27
MgO	5.23	5.27	5.31	5.35
ZnO	4.87	4.88	4.89	4.90
Monogal	5.18	5.24	5.33	5.44
Al_2_O_3_	5.03	5.08	5.13	5.16
TiO_2_	5.51	5.53	5.54	5.56
Untreated carbon fibers	4.45	4.48	4.50	4.52
Oxidized carbon fibers	4.49	4.54	4.59	4.64

**Table 19:** Values of the ($-∆G_{a}^{sp}\left(T\right)\;in\;kJ/mol$) of the dichloromethane and the ethyl acetate adsorbed on the different solid materials at various temperatures.

**(** $-∆G_{a}^{sp}\left(T\right)\;in\;kJ/mol$ **) of Dichloromethane**				
T(K)	323.15	343.15	363.15	383.15
SiO_2_	22.49	21.846	21.269	20.716
MgO	3.312	3.786	4.532	5.211
ZnO	2.449	1.9151	1.2231	0.632
Monogal	2.354	1.965	1.426	0.854
Al_2_O_3_	6.751	6.654	6.575	6.648
TiO_2_	2.546	1.924	1.254	0.723
Untreated carbon fibers	4.096	3.645	3.129	2.548
Oxidized carbon fibers	10.546	9.952	9.379	8.8
**(** $-∆G_{a}^{sp}\left(T\right)\;in\;kJ/mol$ **) of ethyl acetate**				
T(K)	323.15	343.15	363.15	383.15
SiO_2_	4.566	4.015	3.53	3.079
MgO	6.224	7.62	9.112	10.523
ZnO	3.9554	2.7149	1.8004	1.042
Monogal	12.287	9.154	5.642	4.895
Al_2_O_3_	11.624	9.452	7.875	6.125
TiO_2_	3.979	2.417	0.857	-
Untreated carbon fibers	9.500	9.251	9.019	8.975
Oxidized carbon fibers	13.212	12.718	12.242	11.758

**Table 20:** Values of the specific acid and base surface energy contributions $\gamma_{s}^{+}$, $\gamma_{s}^{-}$, $\gamma_{s}^{AB}$ and $\gamma_{s}^{tot.}$ (in mJ/m^2^) of the different solid surfaces.

**Values of** $\gamma_{s}^{+}$ **(in mJ/m^2^)**				
T(K)	323.15	343.15	363.15	383.15
SiO_2_	8.11	6.15	4.66	3.47
MgO	15.07	22.14	31.03	40.57
ZnO	6.08	2.81	1.21	0.40
Monogal	58.72	31.95	11.90	8.78
Al_2_O_3_	52.55	34.06	23.18	13.75
TiO_2_	6.16	2.23	0.27	0.03
Untreated carbon fibers	33.54	31.08	28.63	26.18
Oxidized carbon fibers	64.04	57.33	50.62	43.91
Values of $\gamma_{s}^{-}$ (in mJ/m^2^)				
T(K)	323.15	343.15	363.15	383.15
SiO_2_	275.18	254.53	236.49	219.94
MgO	5.97	7.64	10.74	13.92
ZnO	3.26	1.96	0.78	0.20
Monogal	3.01	2.06	1.06	0.37
Al_2_O_3_	24.80	23.61	22.60	22.65
TiO_2_	3.53	1.97	0.82	0.27
Untreated carbon fibers	8.01	5.99	3.98	1.96
Oxidized carbon fibers	55.89	48.21	40.53	32.85
Values of $\gamma_{s}^{AB}$ (in mJ/m^2^)				
T(K)	323.15	343.15	363.15	383.15
SiO_2_	94.46	79.11	66.37	55.27
MgO	18.96	26.02	36.51	47.52
ZnO	8.91	4.69	1.95	0.57
Monogal	26.61	16.22	7.11	3.62
Al_2_O_3_	65.95	56.00	46.05	36.11
TiO_2_	9.32	4.19	0.95	0.17
Untreated carbon fibers	32.75	27.04	21.32	15.61
Oxidized carbon fibers	119.64	105.11	90.58	76.04
Values of $\gamma_{s}^{tot.}$ (in mJ/m^2^)				
T(K)	323.15	343.15	363.15	383.15
SiO_2_	179.80	146.86	119.23	94.50
MgO	76.31	77.15	81.42	86.23
ZnO	71.12	61.88	54.11	47.71
Monogal	116.87	91.36	67.14	48.53
Al_2_O_3_	128.31	106.07	86.64	67.71
TiO_2_	70.06	60.18	51.74	45.15
Untreated carbon fibers	85.71	74.10	62.49	50.87
Oxidized carbon fibers	171.23	148.53	125.83	103.13

## Data Availability

Data are contained within the article and Supplementary Materials.

## Supplementary Materials

The following supporting information can be downloaded at: https://www.mdpi.com/article/10.3390/molecules29050949/s1, Table S1. Values (in kJ/mol) of London dispersive energy ($-∆G_{a}^{d}\left(T\right)$) of n-alkanes and polar solvents adsorbed on silica particles at different temperatures. Table S2. Values (in kJ/mol) of London dispersive energy ($-∆G_{a}^{sp}\left(T\right)$) of polar solvents adsorbed on silica particles at different temperatures. Table S3. Values (in kJ/mol) of London dispersive energy ($-∆G_{a}^{d}\left(T\right)$) of n-alkanes and polar solvents adsorbed on MgO particles at different temperatures. Table S4. Values (in kJ/mol) of London dispersive energy ($-∆G_{a}^{sp}\left(T\right)$) of polar solvents adsorbed on MgO particles at different temperatures. Table S5. Values (in kJ/mol) of London dispersive energy ($-∆G_{a}^{d}\left(T\right)$) of n-alkanes and polar solvents adsorbed on ZnO particles at different temperatures. Table S6. Values (in kJ/mol) of London dispersive energy ($-∆G_{a}^{sp}\left(T\right)$) of polar solvents adsorbed on ZnO particles at different temperatures. Table S7. Values (in kJ/mol) of London dispersive energy ($-∆G_{a}^{d}\left(T\right)$) of n-alkanes and polar solvents adsorbed on monogal-Zn particles at different temperatures. Table S8. Values (in kJ/mol) of London dispersive energy ($-∆G_{a}^{sp}\left(T\right)$) of polar solvents adsorbed on monogal-Zn particles at different temperatures. Table S9. Values (in kJ/mol) of London dispersive energy ($-∆G_{a}^{d}\left(T\right)$) of n-alkanes and polar solvents adsorbed on alumina particles at different temperatures. Table S10. Values (in kJ/mol) of London dispersive energy ($-∆G_{a}^{sp}\left(T\right)$) of polar solvents adsorbed on alumina particles at different temperatures. Table S11. Values (in kJ/mol) of London dispersive energy ($-∆G_{a}^{d}\left(T\right)$) of n-alkanes and polar solvents adsorbed on TiO_2_ particles at different temperatures. Table S12. Values (in kJ/mol) of London dispersive energy ($-∆G_{a}^{sp}\left(T\right)$) of polar solvents adsorbed on TiO_2_ particles at different temperatures. Table S13. Values (in kJ/mol) of London dispersive energy ($-∆G_{a}^{d}\left(T\right)$) of n-alkanes and polar solvents adsorbed on untreated carbon fibers particles at different temperatures. Table S14. Values (in kJ/mol) of London dispersive energy ($-∆G_{a}^{sp}\left(T\right)$) of polar solvents adsorbed on untreated carbon fibers particles at different temperatures. Table S15. Values (in kJ/mol) of London dispersive energy ($-∆G_{a}^{d}\left(T\right)$) of n-alkanes and polar solvents adsorbed on oxidized carbon fibers at different temperatures. Table S16. Values (in kJ/mol) of London dispersive energy ($-∆G_{a}^{sp}\left(T\right)$) of polar solvents adsorbed on oxidized carbon fibers at different temperatures.
