# Supplementary material for: New Progress on London Dispersive Energy, Polar Surface Interactions, and Lewis’s Acid–Base Properties of Solid Surfaces
Source: Molecules. 2024 Feb 21;29(5):949. doi: 10.3390/molecules29050949 (PMC10933744; doi:10.3390/molecules29050949)
Supplement: Supplementary file 1 [file molecules-29-00949-s001.zip › molecules-2836588-supplementary.pdf]

Supplementary Materials

New Progress on London Dispersive Energy, Polar Surface Interactions, and Lewis's Acid–Base Properties of Solid Surfaces

Tayssir Hamieh[1,2]

[1]Faculty of Science and Engineering, Maastricht University, P.O. Box 616, 6200 MD Maastricht, Netherlands
[2]Laboratory of Materials, Catalysis, Environment and Analytical Methods Laboratory (MCEMA), Faculty of Sciences, Lebanese University, Hadath, Lebanon

Correspondence: Faculty of Science and Engineering, Maastricht University, P.O. Box 616, 6200 MD Maastricht, The Netherlands, E-mail: t.hamieh@maastrichtuniversity.nl

Table S1. Values (in kJ/mol) of London dispersive energy ($-\Delta G_a^d(T)$) of n-alkanes and polar solvents adsorbed on silica particles at different temperatures.

Temperature T (K)	323.15	343.15	363.15	383.15	403.15
C5	19.780	19.780	18.187	16.600	15.444
C6	23.409	23.409	21.524	19.646	18.277
C7	26.534	26.534	24.397	22.269	20.718
C8	30.814	30.814	28.333	25.861	24.060
C9	33.503	33.503	30.805	28.117	26.159
CCl4	24.557	22.516	20.703	18.896	17.580
Nitromethane	16.440	15.074	13.860	12.651	11.770
CH2Cl2	16.229	14.880	13.682	12.488	11.618
CHCl3	19.992	18.330	16.854	15.383	14.312
Diethyl ether	19.744	18.102	16.645	15.192	14.134
THF	17.029	15.613	14.356	13.103	12.191
Ethyl Acetate	19.548	17.923	16.480	15.042	13.994
Acetone	13.404	12.289	11.300	10.314	9.595
Acetonitrile	10.305	9.449	8.688	7.930	7.377
Toluene	23.754	21.779	20.025	18.278	17.005
Benzene	21.295	19.525	17.953	16.386	15.245

Table S2. Values (in kJ/mol) of London dispersive energy ($-\Delta G_a^{sp}(T)$) of polar solvents adsorbed on silica particles at different temperatures.

**Temprature T(K)**	**323.15**	**343.15**	**363.15**	**383.15**	**403.15**
CCl4	6.752	6.810	6.881	6.968	7.129
Nitromethane	13.573	12.367	11.273	10.191	9.378
CH2Cl2	22.490	21.846	21.269	20.716	20.287
CHCl3	19.752	19.304	18.925	18.546	18.250
Diethyl ether	26.838	25.462	23.802	22.314	20.676
THF	35.506	32.787	30.435	27.908	25.593
Ethyl Acetate	4.566	4.015	3.530	3.079	2.732
Acetone	10.612	9.608	8.703	7.816	7.144
Acetonitrile	16.734	15.304	14.016	12.738	11.793
Toluene	17.330	16.724	16.168	15.598	15.187
Benzene	5.640	5.170	4.745	4.328	4.026

Table S3. Values (in kJ/mol) of London dispersive energy ($-\Delta G_a^{d}(T)$) of n-alkanes and polar solvents adsorbed on MgO particles at different temperatures.

Temperature (K)	323.15	343.15	363.15	383.15
C6	19.996	19.127	18.299	17.531
C7	22.673	21.688	20.748	19.877
C8	26.336	25.192	24.100	23.089
C9	28.638	27.394	26.207	25.107
CH2Cl2	12.689	12.138	11.612	11.125
CHCl3	15.630	14.951	14.303	13.703
Diethyl ether	15.479	14.806	14.165	13.570
THF	13.353	12.773	12.220	11.707
Ethyl Acetate	15.313	14.648	14.013	13.425
Acetone	10.505	10.048	9.613	9.210
Acetonitrile	8.048	7.699	7.365	7.056
Toluene	18.645	17.835	17.062	16.346

Table S4. Values (in kJ/mol) of London dispersive energy ($-\Delta G_a^{sp}(T)$) of polar solvents adsorbed on MgO particles at different temperatures.

T(K)	**323.15**	**343.15**	**363.15**	**383.15**
CH2Cl2	3.312	3.786	4.532	5.211
CHCl3	5.833	2.693	1.560	2.176
Diethyl ether	14.415	16.559	18.671	20.721
THF	23.053	25.004	26.928	28.797
Ethyl Acetate	6.224	7.620	9.112	10.523
Acetone	15.723	20.520	25.354	30.243

Acetonitrile	24.370	26.000	27.633	29.269
Toluene	10.771	10.471	10.192	9.945

Table S5. Values (in kJ/mol) of London dispersive energy ($-\Delta G_a^d(T)$) of n-alkanes and polar solvents adsorbed on ZnO particles at different temperatures.

**T(K)**	323.15	343.15	363.15	383.15
C6	12.6777	11.3464	10.2409	9.2860
C7	14.4122	12.8988	11.6421	10.5565
C8	16.7694	15.0085	13.5462	12.2831
C9	18.2572	16.3401	14.7481	13.3728
DCM	7.9317	7.0988	6.4071	5.8097
TCM	9.7642	8.7389	7.8875	7.1520
Diethyl ether	9.8951	8.8560	7.9932	7.2478
THF	8.5518	7.6537	6.9080	6.2639
Ethyl Acetate	9.7236	8.7025	7.8546	7.1222
Benzene	10.7177	9.5922	8.6577	7.8504

Table S6. Values (in kJ/mol) of London dispersive energy ($-\Delta G_a^{sp}(T)$) of polar solvents adsorbed on ZnO particles at different temperatures.

T(K)	**323.15**	**343.15**	**363.15**	**383.15**
DCM	2.449	1.915	1.223	0.632
TCM	1.151	1.061	0.999	0.933
Diethyl ether	7.721	7.045	6.594	6.037
THF	13.596	12.901	12.294	11.517
Ethyl Acetate	3.955	2.715	1.800	1.042
Benzene	0.870	0.690	0.537	0.354

Table S7. Values (in kJ/mol) of London dispersive energy ($-\Delta G_a^d(T)$) of n-alkanes and polar solvents adsorbed on monogal-Zn particles at different temperatures.

T (K)	323.15	343.15	363.15	383.15
C6	25.249	23.667	21.355	18.899
C7	28.600	26.809	24.189	21.408
C8	33.199	31.119	28.078	24.850
C9	36.084	33.823	30.518	27.009
DCM	16.113	15.103	13.628	12.061
TCM	16.610	15.570	14.048	12.433
Diethyl ether	15.260	14.304	12.906	11.422
THF	15.156	14.206	12.818	11.344

Ethyl Acetate	19.324	18.114	16.344	14.464
Acetone	15.412	14.447	13.035	11.536
Acetonitrile	17.138	16.064	14.494	12.828
Toluene	14.696	13.775	12.429	11.000

Table S8. Values (in kJ/mol) of London dispersive energy ($-\Delta G_a^{sp}(T)$) of polar solvents adsorbed on monogal-Zn particles at different temperatures.

T(K)	**323.15**	**343.15**	**363.15**	**383.15**
DCM	2.354	1.965	1.426	0.854
TCM	15.001	11.698	7.938	6.927
Diethyl ether	17.481	15.950	14.408	12.982
THF	23.786	21.503	19.298	17.285
Ethyl Acetate	12.287	9.154	5.642	4.895
Acetone	22.779	20.603	18.500	16.582
Acetonitrile	19.104	16.905	14.694	12.454
Toluene	22.246	20.175	18.138	16.267

Table S9. Values (in kJ/mol) of London dispersive energy ($-\Delta G_a^{d}(T)$) of n-alkanes and polar solvents adsorbed on alumina particles at different temperatures.

T (K)	323.15	343.15	363.15	383.15
C6	21.488	20.220	19.103	18.422
C7	24.393	22.954	21.685	20.912
C8	28.356	26.682	25.208	24.310
C9	30.852	29.030	27.427	26.449
CCl4	20.481	19.272	18.207	17.559
CH2Cl2	13.549	12.749	12.044	11.615
CHCl3	16.684	15.699	14.832	14.303
Ether	16.696	15.710	14.842	14.313
THF	14.415	13.564	12.815	12.358
Ethyl acetate	16.467	15.495	14.639	14.117
Toluene	20.200	19.008	17.958	17.318

Table S10. Values (in kJ/mol) of London dispersive energy ($-\Delta G_a^{sp}(T)$) of polar solvents adsorbed on alumina particles at different temperatures.

T(K)	**323.15**	**343.15**	**363.15**	**383.15**
CCl4	6.751	6.654	6.575	6.648
CH2Cl2	38.808	36.648	34.670	32.613
CHCl3	18.559	16.226	14.028	12.322
Ether	41.085	39.144	37.268	35.790

THF	40.532	38.377	36.371	34.878
Ethyl acetate	11.624	9.452	7.875	6.125
Toluene	18.821	17.490	16.418	15.980

Table S11. Values (in kJ/mol) of London dispersive energy ($-\Delta G_a^d(T)$) of n-alkanes and polar solvents adsorbed on TiO_2 particles at different temperatures.

T (K)	**313.15**	**333.15**	**353.15**	**373.15**
C6	21.431	21.007	20.582	20.165
C7	24.274	23.793	23.313	22.841
C8	28.176	27.618	27.060	26.512
C9	30.623	30.017	29.410	28.815
CH2Cl2	13.681	13.410	13.139	12.873
CHCl3	16.855	16.521	16.187	15.859
THF	14.252	13.970	13.688	13.410
Ethyl Acetate	16.401	16.077	15.752	15.433
Acetone	11.233	11.010	10.788	10.569
Benzene	17.813	17.460	17.108	16.761
Nitromethane	13.847	13.573	13.299	13.030
Acetonitrile	8.711	8.539	8.366	8.197

Table S12. Values (in kJ/mol) of London dispersive energy ($-\Delta G_a^{sp}(T)$) of polar solvents adsorbed on TiO_2 particles at different temperatures.

T(K)	**313.15**	**333.15**	**353.15**	**373.15**
CH2Cl2	2.546	1.924	1.254	0.723
CHCl3	3.146	2.019	0.893	
THF	7.620	6.620	5.620	4.620
Ethyl Acetate	3.979	2.417	0.857	-0.710
Acetone	5.776	4.068	2.362	0.651
Benzene	5.564	4.199	2.834	1.463
Nitromethane	10.394	9.024	7.657	6.283
Acetonitrile	4.615	2.524	0.433	

Table S13. Values (in kJ/mol) of London dispersive energy ($-\Delta G_a^d(T)$) of n-alkanes and polar solvents adsorbed on untreated carbon fibers particles at different temperatures.

T (K)	**313.15**	**333.15**	**353.15**	**373.15**
C6	20.723	19.965	19.206	18.959
C7	23.452	22.594	21.736	21.456
C8	27.206	26.211	25.215	24.891
C9	29.558	28.476	27.395	27.042
CCl4	20.133	19.396	18.659	18.419

CH2Cl2	13.291	12.805	12.318	12.160
CHCl3	16.378	15.779	15.179	14.984
Ether	15.944	15.361	14.777	14.587
THF	13.736	13.234	12.731	12.567
C6H6	17.158	16.530	15.902	15.697
Ethyl acetate	15.851	15.271	14.691	14.502
Acetone	10.842	10.445	10.048	9.919

Table S14. Values (in kJ/mol) of London dispersive energy ($-\Delta G_a^{sp}(T)$) of polar solvents adsorbed on untreated carbon fibers particles at different temperatures.

T(K)	313.15	333.15	353.15	373.15
CCl4	1.723	1.956	2.203	2.518
CH2Cl2	4.096	3.645	3.129	2.548
CHCl3	14.829	13.537	11.761	8.193
Ether	2.112	1.633	1.131	0.546
THF	11.852	11.079	10.310	9.748
C6H6	8.577	8.315	8.055	8.011
Ethyl acetate	9.500	9.251	9.019	8.975
Acetone	10.723	10.282	9.865	9.647

Table S15. Values (in kJ/mol) of London dispersive energy ($-\Delta G_a^{d}(T)$) of n-alkanes and polar solvents adsorbed on oxidized carbon fibers at different temperatures.

T (K)	**313.15**	**333.15**	**353.15**	**373.15**
C6	20.674	19.429	18.209	16.997
C7	23.396	21.988	20.607	19.236
C8	27.142	25.507	23.906	22.315
C9	29.488	27.712	25.972	24.244
CCl4	20.085	18.875	17.690	16.513
CH2Cl2	13.260	12.461	11.679	10.902
CHCl3	16.339	15.355	14.391	13.433
Ether	15.906	14.948	14.010	13.077
THF	13.704	12.879	12.070	11.267
C6H6	17.117	16.086	15.076	14.073
Ethyl acetate	15.814	14.862	13.928	13.001
Acetone	10.816	10.165	9.526	8.892

Table S16. Values (in kJ/mol) of London dispersive energy ($-\Delta G_a^{sp}(T)$) of polar solvents adsorbed on oxidized carbon fibers at different temperatures.

T(K)	313.15	333.15	353.15	373.15
CCl4	2.785	2.843	2.911	2.974
CH2Cl2	10.546	9.952	9.379	8.800
CHCl3	12.788	12.228	11.685	11.134
Ether	7.399	6.965	6.548	6.124
THF	17.020	15.878	14.753	13.623
C6H6	10.429	9.943	9.473	8.995
Ethyl acetate	13.212	12.718	12.242	11.758
Acetone	17.928	16.999	16.094	15.183
